# Monitoring and Surveillance of Aerial Mycobiota of Rice Paddy through DNA Metabarcoding and qPCR

**DOI:** 10.3390/jof6040372

**Published:** 2020-12-17

**Authors:** Sara Franco Ortega, Ilario Ferrocino, Ian Adams, Simone Silvestri, Davide Spadaro, Maria Lodovica Gullino, Neil Boonham

**Affiliations:** 1 Centre of Competence for the Innovation in the Agro-Environmental Sector—AGROINNOVA, University of Turin, Via Paolo Braccini 2, I-10095 Grugliasco (TO), Italy; sarafrancoortega@gmail.com (S.F.O.); marialodovica.gullino@unito.it (M.L.G.); 2 Department of Agricultural, Forestry and Food Sciences (DiSAFA), University of Torino, Via Paolo Braccini 2, I-10095 Grugliasco (TO), Italy; ilario.ferrocino@unito.it; 3 FERA, National Agri-Food Innovation Campus, Sand Hutton, York YO41 1LZ, UK; ian.adams@fera.co.uk; 4 Ente Nazionale per la Risicultura (ENTERISI), Strada per Ceretto 4, 27030 Castello d’Agogna (PV), Italy; s.silvestri@enterisi.it; 5 School of Natural and Environmental Sciences, Newcastle University, Newcastle upon Tyne NE1 7RU, UK; neil.boonham@newcastle.ac.uk

**Keywords:** airborne, spore trap, DNA metabarcoding, rice, *Pyricularia*, *Bipolaris*, ITS

## Abstract

The airborne mycobiota has been understudied in comparison with the mycobiota present in other agricultural environments. Traditional, culture-based methods allow the study of a small fraction of the organisms present in the atmosphere, thus missing important information. In this study, the aerial mycobiota in a rice paddy has been examined during the cropping season (from June to September 2016) using qPCRs for two important rice pathogens (*Pyricularia oryzae* and *Bipolaris oryzae*) and by using DNA metabarcoding of the fungal ITS region. The metabarcoding results demonstrated a higher alpha diversity (Shannon–Wiener diversity index H′ and total number of observed species) at the beginning of the trial (June), suggesting a higher level of community complexity, compared with the end of the season. The main taxa identified by HTS analysis showed a shift in their relative abundance that drove the cluster separation as a function of time and temperature. The most abundant OTUs corresponded to genera such as *Cladosporium*, *Alternaria, Myrothecium*, or *Pyricularia*. Changes in the mycobiota composition were clearly dependent on the average air temperature with a potential impact on disease development in rice. In parallel, oligotyping analysis was performed to obtain a sub-OTU identification which revealed the presence of several oligotypes of *Pyricularia* and *Bipolaris* with relative abundance changing during monitoring.

## 1. Introduction

Airborne microbes are strong contributors to the global ecosystem. Despite the high concentrations of these microbes in the atmosphere [1,2,3], this microbiota has been understudied in comparison with those present in other environments, such as soil or rhizosphere [4]. Airborne organisms play a key role in atmospheric processes, such as aerosol particles for cloud formation, ice nucleation, rainfall cycles [5,6,7], and within the biogeochemical cycles of the earth [8]. In addition, the airborne microbiota is important in disease transmission, by contributing to both long and short distance spread of diseases [9,10]. Different studies regarding the aerial microbiota in suburban and city regions [11,12,13], as well as in indoor spaces [14], have been performed in order to monitor important organisms that may affect human health and it has been confirmed that between 4–11% of the airborne fine particle mass (≤2 µm diameter) is made up of fungal spores [15].

Rice (*Oryza sativa* L.) is a monocotyledonous annual grass and the most important staple food crop globally, providing approximately 50–80% of the diary calories intake [16] for over 50% of the world population [17]. Global rice production exceeded 769 million tons in 2018 [18], with Asia being the most important rice-growing and consuming continent, contributing to more than 90% of the world production. The European Union is a relatively small rice producer (2.9 million tonns of rice produced in 2018). This crop is mainly cultivated in Mediterranean countries, such as Italy (50% of the EU production), Spain, Portugal, and France.

Rice production increased due to the improvements in cultivation practices and the use of high-yield hybrid cultivars [19]. However, this crop is susceptible to many biotic diseases, with over 100 fungal pathogen species isolated from rice [20] and, in global terms, rice diseases cause approximately 10–15% of the yield losses [21,22].

Rice blast, caused by *Pyricularia oryzae* Cavara, and rice brown spot, caused by *Bipolaris oryzae* (Breda de Haan) Shoemaker, are two of the most important rice diseases affecting the crop during all growth stages [20]. Both pathogens have a polycyclic life cycle, with primary and secondary airborne inoculum playing an important role in dissemination [23]. *P. oryzae* overwinters in infected crop debris and seeds on the soil forming the primary inoculum, ascospores, which are released and spread over long distances. When ascospores are deposited on new leaves, they may germinate and penetratate the host cells forming hyphae and causing tissue wilting. Local spread in the plants results in the eruption of sporulating lesions, which decrease the photosynthetic area of the plant and thus depress yield. Conidia are released from pycnidia formed on the lesions and are transmitted by splashes over shorter distances. Agronomical practices can result in a significant decrease in rice blast incidence [24]. Rice blast and brown spot management is based on the use of resistant cultivars, timely application of fungicides, monitoring of the pathogen occurrence [25] and good agricultural practices to avoid the overwintering-inoculum on the debris.

*B. oryzae*, agent of brown spot, overwinters in the seeds and stubbles, causing the primary infection by airborne conidia in the nursery and in the main field [23]. The spores germinate and enter the seedling roots or the coleoptile. The release of new spores formed during rice growth causes secondary infections and therefore the characteristic leaf spots. These spores can infect leaves or panicles, and later the grains, thus closing the life cycle.

For both pathogens, the conidia release into the air is governed by meteorological parameters and is synchronised with the crop phenological stage of the plant. Airborne spores follow the air movement model, including pre-conditioning, take-off and ascent, horizontal transport, descent and landing, and impact [26], which also depends on the availability of plant surfaces. Meteorological parameters, such as temperature and rainfalls, humidity, as well as agronomical practises (e.g., volume of water for irrigation and amount of nitrogen for fertilization), play a decisive role in the quantity of released spores and the number of cycles of both pathogens during the year. Both diseases are more severe during periods of warm temperatures and high humidity. However, seasonal variations in the aerosolized microorganisms have been reported [27,28] and therefore changes in the quantity of spores released are difficult to predict. Forecasting models to predict the percentage of infected leaf area caused by rice blast and brown spot diseases are based on weather conditions that affect the airborne spores [29,30,31]. In addition, the data obtained from the identification and quantification of pathogen airborne spores collected in spore traps that are routinely used in rice growing regions, including northern Italy, are used as a surveillance tool for *P. oryzae*, *B. oryzae*, or *Fusarium fujikuroi* [23,32]. 

These air samplers move particulate matter from the air onto collection surfaces, either in an active or passive way and have been used to monitor fungal spores for agricultural applications since the 1950s [33]. The most commonly used spore traps are Hirst types [34] which deposit spores onto sticky tapes on rotating drums enabling temporal measurements of spores as the tape rotates, exposing different zones to the airflow. Rotarods are the simplest samplers, using small electric motors to spin sticky collection rods in the air, which are changed periodically to provide temporal data traditionally, microscopy is used quantification of air-disseminated conidia on spore trapping tape [35]. However, the procedure is long and tedious and requires an experienced operator to recognize the conidia by their microscopic features. Furthermore, several drawbacks have been reported due to the high concentration of spores in some areas of the tapes that impede the microscope-based quantification and the distinction among the different conidia. In addition, different operators can result in variations in the count of fungal spores, and the error may be increased if only partial tapes are counted under the microscope [36]. The presence of plant material, insects, dust and other particles can be also a problem during the observation of the tapes. DNA based techniques have overcome many of the problems caused by traditional identification methods [37] and have allowed a rapid identification of spores from spore trapping devices. However, the adhesive used for the capture of the spores in the spore trapping tapes may be an inconvenience during the DNA extraction causing inhibition of the reaction and therefore an underestimation of the amount of the pathogen [38].

Spore trapping and amplicon sequencing have been recently used in combination to study airborne fungi, including plant pathogens for surveillance purposes [39,40,41,42].

Understanding the airborne microbial component, taking into account not only the amount of pathogenic spores, but also interactions and dynamics with other microorganisms, and with meteorological data may improve the ability of disease forecasting tools to anticipate changes in the habitat and therefore provide a more suitable prediction, guiding interventions in the management of the crop (e.g., application of fungicides) only when pathogen is present and disease conducive conditions occur. The objective of this study was to use the DNA metabarcoding for monitoring *P. oryzae* and *B. oryzae* in aerial fungal spore captures, compared to the use of qPCR (based on TaqMan chemistry). In addition, the aerial mycobiota of a rice crop was explored to investigate changes in the community as a function of seasonal changes in meteorological conditions. 

## 2. Materials and Methods

### 2.1. Sample Preparation

A volumetric spore sampler (Burkard Manufacturing Co. Ltd., Rickmansworth, Hertfordshire, UK) was placed in a field of rice ‘Vialone Nano’ in Castello d’Agogna (Lombardy, Italy) belonging to Ente Nazionale per la Risicoltura (45.248696, 8.699970). The spore sampler was used throughout the growing season (25/06/2016–12/09/2016). The growth stages of rice during the trial are included in Table S1. Air was sampled at a rate of 10 L/min and the spores were collected on tapes coated in sterile silicone. Sampling orifices were horizontal and approximately 1.5 m above ground level. Each week, the sticky tapes were replaced and the used ones were cut into 7 single day pieces with sterile scissors and stored in 50 mL polypropylene tubes at −20 °C until DNA extraction. Meteorological data, including daily average temperature, relative humidity, rainfall, average wind speed and wind direction were recorded in a closed meteorological station present at the same location. Average daily temperatures were collapsed into quartiles as follows: quartile T1 corresponds to temperatures ranging from 19.7 °C to 22.5 °C, T2 from 22.5 °C to 23.7 °C, T3 from 23.8 °C to 24.5 °C, and T4 from 24.6 °C to 26.1 °C. Similarly, also the wind speed and relative humidity (RH) were collapsed into quartiles (Table S2) (for wind speed, first quartile was set from 1.1 to 1.3 m/s. second quartile from 1.3 to 1.6 m/s, third quartile from 1.61 to 1.9 m/s, and forth quartile from 1.91 to 2.9 m/s; whilst for RH, the first quartile was set from 51 to 70%, second from 70.1% to 74%, third from 74.1 to 77% and forth from 77.1 to 91%).

### 2.2. Disease Index

The symptoms of rice blast, caused by *P. oryzae*, were grey-green and/or water-soaked lesions with a darker green border that later expanded rapidly to several centimetres in length becoming lighter in colour with a distinct necrotic border. Symptoms on leaves were evaluated as follows: (0) no symptoms; (1) small brown spots on the upper leaves; (2) small round spots and slightly elongated or grey necrotic spots of 1–2 mm in diameter with a distinct brown margin; (3) spots as described in 2 but with a significant number of lesions on the upper leaves; (4) lesions with the size of 3 mm or more affecting less than 4% of the leaf surface; (5) lesions affecting 4–10% of the leaf surface; (6) lesions affecting 11–25% of the leaf surface; (7) lesions affecting 26–50% of the leaf surface; (8) lesions affecting 51–75% of the leaf surface and many dead leaves; (9) lesions affecting more than 75% of the leaf surface. Symptoms on the panicles were evaluated as follow: (0) no lesions; (1) panicles affected less than 5%; (3) panicles affected 5–10%; (5) panicles affected 11–25%; (7) panicles affected 26–50%; (9) panicles affected more than 50%. Rice brown spot caused by *Bipolaris oryzae* were also evaluated with a similar disease index. After testing, normality and variance distribution, Kruskal–Wallis test was performed using the package car, with the Dunn test conducted using the dunnTest function of the FSA package in R, in order to associate the meteorological parameters (temperature, average wind speed, relative humidity and wind direction) with the disease index. To perform this analysis the disease index measure at the beginning of each week was considered the same until the next measurement was performed. Plots of the results, showing the Dunn test groups, were obtained.

### 2.3. DNA Extraction from Sticky Trips

CTAB lysis buffer (2 mL) [43] with 1% *v*/*v* antifoam solution and 1% RNase A (Qiagen, Manchester, UK) were added to each daily spore tape. The samples were heated at 60 °C for 30 min, with mixing every 10 min to soften the silicone. A mixture of 2.3 and 0.5 mm zirconia-silica beads (Thistle Scientific, Glasgow, UK) were added to the samples and mixed for 4 min to elute all the spores into the lysis solution. The samples were left to stand for 30 min and the clear lysate was collected and used for gDNA extraction using the NucleoSpin^®^ Plant II kit (Macherey-Nagel, Dueren, Germany) according to the manufacturer’s instructions. DNA was stored at −20 °C.

### 2.4. Real-Time qPCR

Two qPCR with TaqMan assays were used to quantify the spores of *P. oryzae* and *B. oryzae* in the air samples. The *P. oryzae* qPCR assay designed by Su’udi et al. [44] and validated also for a LAMP assay [45] on the *MHP1* gene, encoding an hydrophobin, was performed using the primers MHP1F (5′-TCGATGCCGACAACTTCTCCGA-3′) and MHP1R (5′-ACCCTGGTCAAGCTGTTCGATTGT-3′), and probe (5′-TGCCCATCGTAAGTTCCTTCTTCGCA-3′). *B. oryzae* spores quantification was carried out using the qPCR assay designed by Su’udi et al. [46] based on the gene scytolone dehydratase using primers CmSCD1_44F (5′-CATGTGTGCAGTAAAGTGACTC-3′) and CmSCD1_ 302R (5′-GTCTTGAGGAGGGGGTT-3′) and the probe (5′-ACAAGATATGGGAGGCGATGCCAG-3′). Both probes were 5′ labelled with 6-carboxyfluorescein (FAM) and 3′ labelled with the Black Hole Quencher^®^ (BHQ^®^). The reactions were carried out on a StepOne thermal cycler (ThermoFisher Scientific, Loughborough, UK) using the following cycling conditions: initial denaturation at 95 °C for 4.5 min and 40 cycles (15 s at 95 °C and 15 s at 60 °C). Positive control DNA was extracted from cultured pathogen and a negative control of water was included in each experiment. A standard curve was generated for each assay using 10-fold dilutions of pathogen DNA ranging from 1.37 ng to 1.37 fg for *P. oryzae* and 2.23 ng to 2.23 fg for *B. oryzae.* All the samples and the standard curves were tested in triplicate.

The final number of cells was calculated according to the standard curves, taking into account the elution volume of the DNA extraction (100 µL) and the genome weight of both pathogens (*P. oryzae* = 0.0000378 ng and *B. oryzae* = 0.000034 ng), using the formula: number of cells/µL = DNA quantity/genome weight). 

Wilcoxon rank sum tests were used to find significant differences in the number of cells for both pathogens according to the variables (meteorological parameters and growth stage of the plant). *p*-values were adjusted for multiple testing using the Benjamini–Hochberg procedure, which assesses the false discovery rate (FDR).

### 2.5. ITS Amplicon Target Sequencing

The internal transcribed spacer region (ITS rDNA) was amplified using the primers Ky02F (5′-TAGAGGAAGTAAAAGTCGTAA-3′) and ITS1 Wobble (5′-CWGYGTTCTTCATCGATG-3′) combined with the Illumina overhang adapter sequences. Positive and negative controls were included in each PCR assay. The reaction mixture (35 µL) contained 12.5 µL of the Q5^®^ Hot Start High-Fidelity DNA Polymerase, (New England Biolabs, UK), 0.9 µL of each primer (10 µM), and 1 µL of genomic DNA. The PCR was performed on 2720 Thermal Cycler (ThermoFisher Scientific) using the following cycling conditions: 98 °C for 2 min, followed by 40 cycles of 98 °C for 20 s, 54 °C for 30 s and 72 °C for 90 s with a final extension at 72 °C for 5 min. The amplicon PCR was performed in triplicate and the size of the products were checked using gel electrophoresis. 

The PCR products were tagged using the Nextera XT Index Kit (Illumina, Cambridge, UK) using the following conditions: 95 °C for 3 min, 8 cycles of 95 °C for 30 s, 55 °C for 30 s, 72 °C for 30 s, and a final extension at 72 °C 5 min. The amplicons were purified using 20µl AMPure XP Clean Up beads (Beckman Coulter Ltd., High Wycombe, UK) for each 25 µl of PCR product and quantified using the Bioanalyzer 2001 (Agilent Technologies, Stockport, UK) following the manufacturers protocols. The 4200 TapeStation Instrument (Agilent Technologies, Cheadle, UK) using High Sensitivity D1000 ScreenTape (Agilent Technologies) enabled the measurement of the mean amplicon size, the data was used for the Illumina Calculator software to obtain the volume of each sample needed to obtain a 20 nM pool of all the samples. A 4 nM pool was obtained taking into account the weight of the pool library measured using a Qubit Fluorometric Quantitation (ThermoFisher Scientific), and the mean amplicon size. A denaturised 20 pM pool was obtained by mixing 5 µL of 0.2 N NaOH with 5 µL of the 4 nm pool in 1 mL final volume of the HT1 buffer (Illumina). The final 10 pM library combined with 10% PhiX was run. Sequencing was performed with a MiSeq Illumina instrument using V3 chemistry, generating 250 bp paired end reads.

### 2.6. Bioinformatics and Statistical Analysis

Raw reads were trimming based on quality (Phred scores) using the FastQC toolkit (http://www.bioinformatics.babraham.ac.uk/projects/fastqc/) and Paired-end reads were assembled using FLASH software [47] using default parameters. Combined reads were further quality filtered (at Phred < Q20) using QIIME 1.9.0 software [48]. Briefly, chimeras were removed by using Usearch v8.1 software [49] and reads shorter than 150 bp were discarded by using Prinseq (http://prinseq.sourceforge.net/). OTUs (operational taxonomic units) were picked at 97% similarity by means of UCLUST clustering methods [50], and centroid sequences of each cluster were used to assign taxonomy using the UNITE ITS database by means of the RDP classifier [51]. Statistics and plotting were carried out in the R environment. Alpha diversity indices were calculated using the diversity function of the vegan package [52]. The Shannon–Wiener diversity index *H′*, the chao1, and a total number of observed species were further analysed using the Wilcoxon test to assess differences between months and temperature. In order to avoid bias due to different sequencing depths, all samples were rarefied at 16,024 reads after raw read quality filtering and singleton OTUs were discarded.

A filtered OTU table was generated at 0.2% abundance in at least 20 samples through QIIME and the relative abundance of OTUs from the three replicates from each sample point were averaged. The OTU table displays the highest taxonomy resolution that was reached when the taxonomy assignment was not able to reach the genus level, the family or order was display. The OTU table was used to build a principal component analysis (PCA), after logarithm transformation of the data, as a function of the months or temperature by using the made4 package of R. In addition, redundancy analysis (RDA) was performed after testing the heterogeneity in community composition, with the cca function of the vegan package in R. The rda used the relative abundance of all the OTUs after a Hellinger transformation which was performed using the function decostand of the package vegan in order to calculate a distance matrix that gave low weights to rare species. Afterward, the distance matrix was used to find linear combinations of the meteorological parameters such as relative humidity, rainfall, temperature, wind speed, wind direction and the different growth stages of the rice that explain the distance matrix. Triplot of the meteorological parameters (explanatory variables), weight sum of species (wa) and dates was then plotted with the ggord package in R and the scaling 2 where the angles between vectors represent linear correlations. Correlation between the log-transformed relative abundance of the different OTUs was assessed by using the corr.test function of the psych package with Spearman test and adjusted to assess the False Discovery Rate (FDR). Corrplot function of the corrplot package was used to plot the correlation matrix of the significant correlations (FDR < 0.01). The relative abundance (log transformed) was correlated with the disease index of *Pyricularia* in order to understand if any fungal species may be related to the disease development.

Anosim statistical tests were used to confirm differences among the variables, including time and meteorological parameters. Wilcoxon rank sum tests were used to find significant differences in fungal taxa abundance according to the variables. *p*-Values were adjusted for multiple testing using the Benjamini-Hochberg procedure, which assesses the false discovery rate (FDR). The daily temperature, the wind speed and relative humidity were clustered in quartiles to perform the statistical analysis. Growth stage of the plant was also used to perform Wilcoxon rank sum tests between the relative abundance of the OTUs and the growth stage. The core mycobiota was composed by the OTUs present for at least 20 days at a relative abundance higher than 0.5%.

All the sequencing data were deposited at the Sequence Read Archive of the NCBI (SRA accession number SRP136527).

### 2.7. Oligotyping Analysis

From the total reads, the corresponding rice pathogens *Pyricularia* and *Bipolaris* were extracted and used separately for entropy and oligotyping analysis, as described by Eren et al. [53]. High entropy positions were selected to compute the oligotyping analysis using the C option as follows: *Pyricularia* positions were 27, 30, 33, 42, 45, 48, 58, 59, 65, 67, 75, 82, 85, 86, 87, 115, 131, 132, 141, 143, 145, 164, 188, 194, 206, 209, 210, 213, 215, and *Bipolaris* positions were 27, 29, 30, 48, 70, 107, 130, 143, 152, 161, 168, 174, 187, 200, 208, 263, 264, 266, 272, 277, 278. In all cases, the oligotypes obtained satisfied quality criteria: (i) appearing in at least 10 samples; (ii) occurring in more than 1% of the reads for at least one sample; (iii) representing at least 500 reads taking into account all the samples combined using -M option; (iv) having the most abundant unique sequence with a minimum abundance of 50. RDA using the relative abundance of oligotypes to find correlation with all meteorological parameters and the phenological stages of the plant were also performed as previously described. Pairwise Wilcoxon tests were used in order to determine significant differences in specific oligotype abundance according to samples, in function of growth stage and meteorological parameters as previously explained.

### 2.8. Correlation between Oligotyping Analysis Results and Number of Cells Calculated by qPCR

The correlation between the relative abundance of each single oligotype and the number of spores of *P. oryzae* and *B. oryzae* calculated with the qPCR was checked with cor.test with Spearman method using the stat package in R environment. To perform the analysis, the relative abundance of the oligotypes was multiplied by the relative abundance of the genera to obtain the relative abundance of each oligotype in the total of OTUs.

## 3. Results

### 3.1. Disease Severity

Symptoms of rice blast and brown spot disease were evaluated in the field weekly, starting from 19 July. No symptoms of brown spot disease were observed throughout the trial, whilst symptoms of rice blast were found in both leaves and necks. The first visible symptoms of rice blast were found on leaves after visual examination of July 19th, corresponding with the growth stage BBCH 41, with a disease index of 3.0. The disease severity increased throughout the trial and a disease index of 9.0 was recorded at the end of the trial (Table S3). Kruskal–Wallis test with Dunn test (Supplementary Figure S1) showed that both neck and leaf blast was influenced by the temperature quartile, with significant differences in the disease index between the fourth quartile and the first and second quartiles. These data showed the positive influence of moderate temperatures of around 20 °C (FDR < 0.05).

The wind speed and direction were also critical for the development of the disease (Supplementary Figure S1), with significant differences in the blast disease index between the first and four quartiles, indicating a negative influence of faster winds on the disease and between north-northeast and north-northwest winds. 

However, no significant differences were shown with the relative humidity (FDR > 0.05) (Supplementary Figure S1).

### 3.2. qPCR Assays

*B. oryzae* was first detected on 18 August, corresponding with the growth stage of BBCH 80, and continued to increase during August reaching its maximum level at the end of the trial (7.8 × 10^4^ cells on 11 September) (Supplementary Figure S2A). *P. oryzae* was first detected on 26 June and continued to increase, reaching its highest level in August (9.1 × 10^4^ cells on 23 August, corresponding to growth stage BBCH 80), with a decrease in September (Supplementary Figure S2B). Neither qPCR produced repeatable results, as positive results were recorded in only one or two out of the three technical replicates (colour legend in Supplementary Figure S2). Meteorological parameters did not cause significant differences in the number of cells of any pathogen (FDR < 0.05), however, if the *p*-value was not adjusted to assess the FDR, significant variation was found between the number of cells of *P. oryzae* and the relative humidity. Specifically, there were changes between the first (7.9 × 10^3^ cells) and forth quartile (2.3 × 10^4^ cells) of relative humidity.

The number of cells of *P. oryzae* was positively correlated with the phenological phase of rice with an increase over the growing season. The number of cells increased from 4.8 × 10^2^ cells at tillering (BBCH 21) to 3.6 × 10^4^ cells at early dough (BBCH 80), to back down to 4.5 × 10^3^ cells at full ripening. No statistically significant changes (FDR > 0.05) were found with the number of cells of *B. oryzae* and the growth stage.

### 3.3. Mycobiota Diversity and Core Mycobiota of Air Samples

A total of 9,433,076 raw reads (2 × 300 bp) were obtained. After assembly and quality filtering, a total of 9,110,867 reads were retained, ranging from 16,290 reads/sample (30 August), to 608,084 reads/sample (17 July), with an average of 39,440 reads/sample, and an average sequence length of 262 bp. The rarefaction analysis and the Good’s coverage expressed as a percentage (98%) indicated that there was satisfactory coverage for all the samples (Supplementary Table S4).

**Figure 1:**
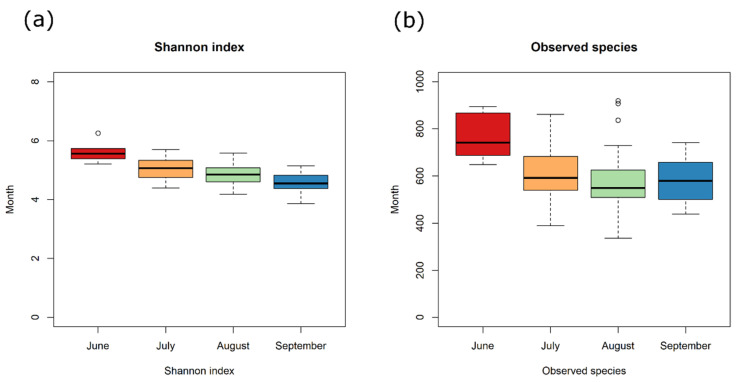
A. Shannon Index (**a**) and number of Observed OTUs (**b**) over time. Samples are labelled according to month (June, July, August, and September). Boxes represent the interquartile range (IQR) between the first and third quartiles, and the line inside represents the median (2nd quartile). Whiskers denote the lowest and the highest values within IQR from the first and third quartiles, respectively. Circles represent outliers beyond the whiskers.

A total of 63 OTUs were identified through the trial. However, the total number of OTUs, as well as the Shannon–Wiener diversity index *H*’ (Figure 1; Supplementary Table S4), clearly showed that there were higher levels of complexity with more OTUs identified at the beginning of the trial (June) compared to the end, with a gradual decrease in community complexity as the growing season progressed (FDR < 0.05).

**Figure 2:**
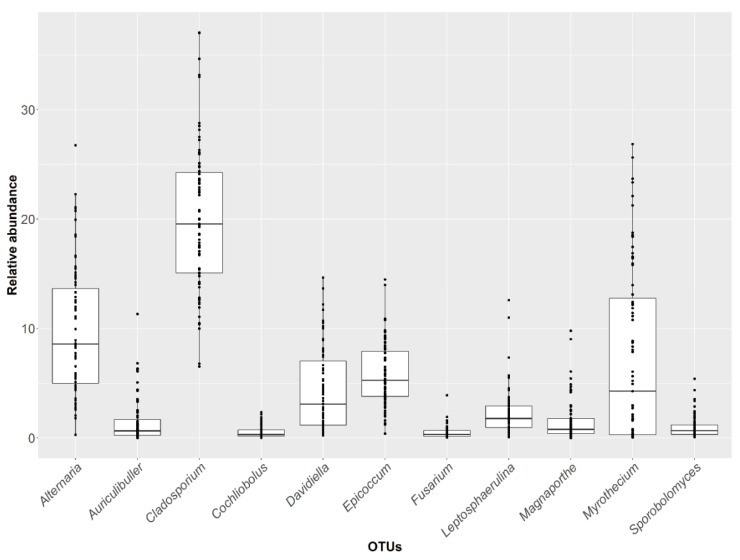
Abundance (%) over time of the 14 genera within the OTU core corresponding to the OTU core identified in 83.5% of the samples. Boxes represent the interquartile range (IQR) between the first and third quartiles, and the line inside represents the median (second quartile). Whiskers denote the lowest and the highest values within 1.56 IQR from the first and third quartiles, respectively. Circles represent outliers beyond the whiskers.

The abundance of the different genera present in the core mycobiota varied according to the time-point sampled. The most abundant OTUs identified until genera were represented using box plots in the Figure 2. *Hyphodontia* and *Coriolopsis* relative abundance were not shown. The other genera show a considerable inter-sample variability during the season. A few OTUs had a median relative abundance higher than 4% throughout the experiment, including *Cladosporium* (20.2%), *Alternaria* (9.9%), *Epicoccum* (5.8%), *Myrothecium* (7.3%), and *Davidiella* (4.3%) (Supplementary Table S5).

### 3.4. Mycobiota Development

**Figure 3:**
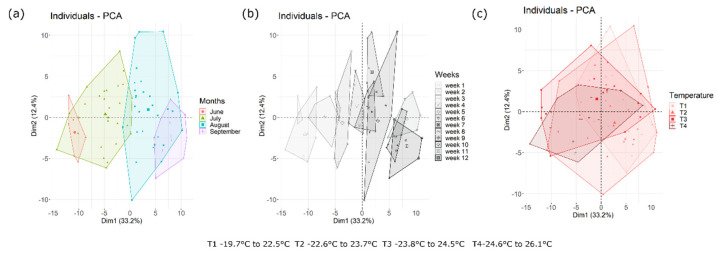
Principal component analysis (PCA) based on OTU table. Samples are grouped according to month (**a**), week (**b**) or temperature (**c**). Week 1 and 2 correspond with the period between 25 June 2016 to 4 July 2016, weeks 3, 4 and 5 correspond with the period between 4 July 2016 to 24 July 2016, weeks 6, 7, 8 and 9 correspond with the period 24 July 2016 to 22 August 2018, whilst the last three weeks are from 22 August 2016 to 12 September 2016. The first component (horizontal) accounts for 33.2% of the variance and the second component (vertical) accounts for 12.4%.

Taking into account the sampling time-point (day or week) and the quartile of average daily temperature range, some differences were observed in OTU abundance. Differences between time or temperature quartiles were further demonstrated by principal component analysis (PCA) based on the relative abundance of the main OTUs (Figure 3). The PCA clearly showed a shift in the mycobiota composition across time (Figure 3a,b), a gradient of separation of the mycobiota was observed from the beginning to the end of the trial. Taking into the account the shift in the average temperature during the trial (quartiles of the temperature range from 19.7 °C to 26.1 °C), it was possible to observe (Figure 3c) that the samples from the fourth quarter (24.6 °C to 26.1 °C) were well separated from the earlier time points (FDR < 0.05). Anosim statistical tests based on OTU tables were also used to confirm significant differences (FDR < 0.05) with time, temperature and wind speed. No statistical differences (FDR > 0.05) were observed based on relative humidity or rainfall with this analysis.

RDA was performed in order to obtain a general idea of the importance of the all the environmental variables and the phenological stage in the matrix. A total of 55.9% of the data was explained by these variables, however this value might be biased, and it was adjusted for the number of the environmental variables, obtaining a value of 44.6%. After plotting the scaling 2 of the redundancy analysis, which shows a linear correlation between all angles, we observed differences in the behaviour of all the OTUs after correlating with environmental parameters and phenological stages (Supplementary Figure S3). *Pyricularia* relative abundance was highly influenced by the wind direction and high relative humidity and low rainfall and the boot stage, flag leaf opening and panicle emerging stages and less influenced by temperatures. On the other hand, the wind direction north-northwest correlated positively with the relative abundance of *Myrothecium* spp. and *Auriculibuller* spp. The abundance of these two OTUs was also positively correlated with the end the trial corresponding to the early dough and fully ripe phenological stages. The low temperatures and moderate wind speed and the medium RH also increased the relative abundance of *Myrothecium* spp. and *Auriculibuller* spp. Cladosporium was correlated with high temperatures and high wind speed, whilst other genera belonging to the OTU core (*Epicoccum*, *Coriolopsis*, *Fusarium*, *Alternaria*, *Bipolaris* and *Davidiella*) were influenced positively by low relative humidity, moderate to high temperature and high wind speed. 

**Figure 4:**
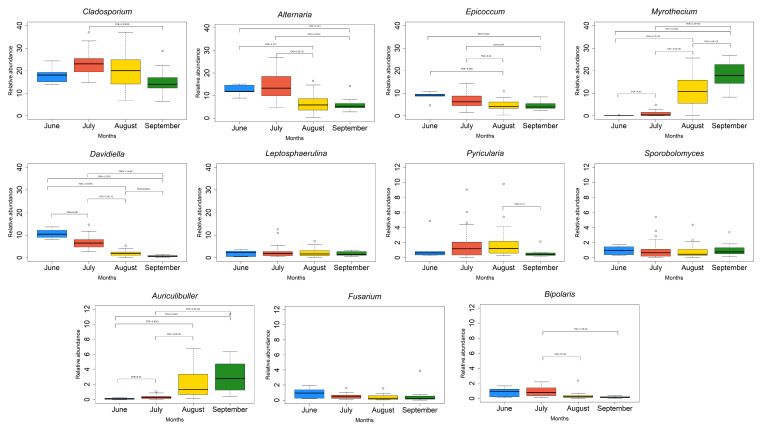
Abundance (%) of the OTUs belonging to the OTU core significantly different (FDR < 0.05) based on the sampling month (June, July, August and September). Note the different scales on the relative abundance.

As the RDA, only explained approximate 45% of the data, we focused on the individual variables. Regarding the development of the mycobiota across the months, significant differences in the OTU relative abundances (FDR < 0.05) were observed for each month (Figure 4). It was possible to observe a decrease from June to September (FDR < 0.05) of *Cladosporium* (from 18.2% in June to 15.2% in September, with maximum levels in July (23.1%) and August (19.8%). *Alternaria* decreased from 12.4% in June to 6.0% in September, passing through 14.0% in July and 6.9% in August. *Epicoccum* decreased from 8.7% to 4.7%, with 6.8% in July and 4.7% in August. *Davidiella* decreased significantly from 10.6% in June to 6.9% in July, to 1.98% in August and 0.73% in September. *Pyricularia* passed from 1.3% in June to 0.6% in September, with maximum levels of 1.8% in July and August. *Bipolaris* decreased constantly, from 0.9% in June to 0.2% in September. Other groups, such as *Aspergillus, Penicillium*, and Sclerotiniaceae, also showed a decreasing level of relative abundance from June to September with a peak in the middle of the trial.

The opposite was observed for *Myrothecium* that significantly increased (FDR < 0.05) from 0.2% in June, to 0.93% in July, 10.5% in August, and 18% in September, and *Auriculibuller*, from 0.12% in June to 0.3% in July, 2.1% in August, reaching 3.5% in September. The relative abundance of some genera such as *Leptosphaerulina*, *Sporobolomyces*, and *Fusarium* did not significantly change throughout the season (FDR > 0.05).

**Figure 5:**
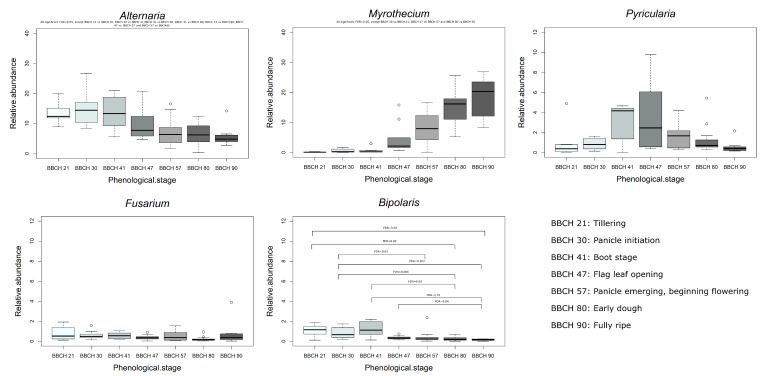
Abundance (%) of *Alternaria, Myrothecium, Pyricularia, Fusarium* and *Bipolaris* clustered (FDR < 0.05) among the growth stages (BBCH: 21, tillering; 30, panicle initiation; 41, boot stage; 47, leaf opening; 57, panicle emerging; 80, early dough; and 90, fully ripening).

Among the OTUs core, some genera varied in a significant way according to the phenological stage of rice (Figure 5), including *Bipolaris* (1.0%, 0.8%, 1.3%, 0.4%, 0.4%, 0.3%, 0.1%); *Myrothecium* (0.1%, 0.6%, 0.7%, 4.7%, 7.9%, 14.7%, 14%); *Alternaria* (13.9%, 14.8%, 13.8%, 9.6%, 7.4%, 6.6%, 5.9%); *Cladosporium* (18.8%, 21.9%, 27.7%, 9.9%, 21.1%, 18.4%, 11.9%); *Epicoccum* (8.1%, 6.2%, 8.7%, 5%, 4.4%, 5.4%, 3.2%); *Davidiella* (10.5%, 7.3%, 6.1%, 4.6%, 2.5%, 1.1%, 0.6%); *Auriculibuller* (0.1%, 0.4%, 0.2%, 0.8%, 1.9%, 3%, 6.2%). *Fusarium* and *Pyricularia* did not show significant differences when correlated with the phenological stages (FDR > 0.05), but if the *p*-value was not adjusted to test the FDR, there were significant differences between some phenological stages in the relative abundance of *Pyricularia*, that was higher during the boot stage and flag leaf opening stage (data not shown). Differences also were found in *Fusarium* relative abundance in function of the phenological stage if the *p*-value (*p*-value < 0.05) was not adjusted to the FDR, especially between the early dough with the tillering, panicle initiation, and boot stage (data not shown).

**Figure 6:**
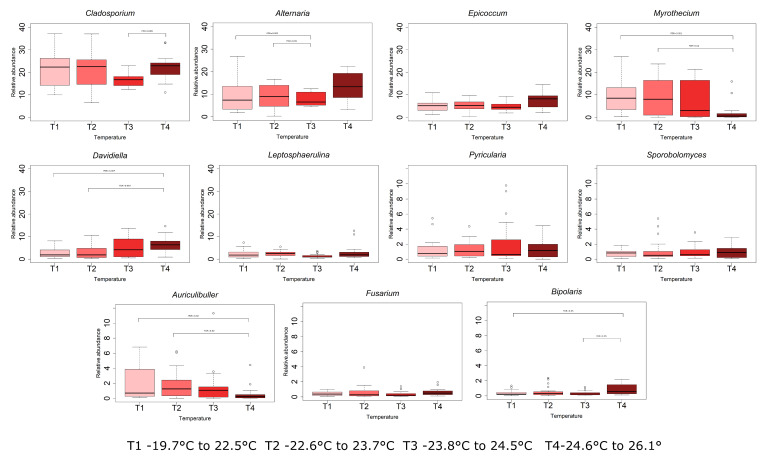
Abundance (%) of the OTUs belonging to the OTU core significantly different (FDR < 0.05) among the temperature quartiles T1 corresponding to average daily temperatures ranging from 19.7 °C to 22.5 °C, T2 from 22.5 °C to 23.7 °C, T3 from 23.8 °C to 24.5 °C and T4 from 24.6 °C to 26.1 °C. Note the different scales on the relative abundance.

By considering the variation of the mycobiota as a function of the shift in average daily temperature, it was possible to find a specific correlation between temperature and OTUs. In detail (Figure 6), with FDR < 0.05, *Alternaria* (from 9.05% in T1 to 11.2% in T4), *Epicoccum* (from 5.0% in T1 to 7.6% in T4) *Davidiella* (from 2.8% in T1 to 7.6% in T4), and *Bipolaris* (from 0.4% in T1 to 0.9% in T4) were favoured by higher average daily temperature. Other genera, such as *Ustilago*, became more abundant with higher temperatures (from 0.2% in T1 to 0.5% in T4). Conversely, *Myrothecium* (from 9.6% in T1 to 2.1% in T4), *Auriculibuller* (from 2.0% in T1 to 0.6% in T4), and *Plectosphaerella* (from 0.2% in T1 to 0.1% in T4) were favoured by lower temperature.

Relative humidity and wind speed caused changes in the mycobiota composition although not many significant differences between the most abundant OTUs and the relative humidity were found. Only *Epicoccum* was favoured by higher relative humidity, with significant differences between the second quartile and the fourth (relative abundance from 5.75 to 6.62%) and from the third to the fourth (relative abundance from 4.12 to 6.62%). Other lower abundant OTUs such as *Monographella* and *Pleospora* were negatively influenced by higher relative abundance. Wind speed did not correlate significantly with any OTU.

The OTU co-occurrence/exclusion pattern is shown in Supplementary Figure S4, where only significant correlations are reported (FDR < 0.001). Regarding the most abundant OTUs, *Myrothecium*, *Alternaria*, and *Auriculibuller* displayed the highest number of negative correlations and exclusion patterns. *Myrothecium* showed a strong exclusion pattern with *Alternaria*, *Chalastospora*, and *Davidiella*, while a positive co-occurrence pattern was observed with *Auriculibuller* and *Dissoconium.* Whilst *Auriculibuller* displayed a negative correlation with *Chalastospora*, *Davidiella*, and *Ustilago.* Strong co-occurrence was shown between *Alternaria* and *Chalastospora*, *Bipolaris*, and *Davidiella*, between *Aspergillus* and *Penicillium*, between *Phellinus* and *Hyphodonthia*, and between *Chalastospora*, *Bipolaris* and *Davidiella*. No correlation pattern was observed for important rice pathogens, such as *Bipolaris*, *Fusarium*, and *Pyricularia* (FDR > 0.01). Interestingly, some OTUs, such as *Myrothecium*, were positively correlated (FDR < 0.01) to the development of *Pyricularia* symptoms (leaves and neck), whilst other OTUs (e.g., *Davidiella*) were negatively correlated (Supplementary Figure S5).

### 3.5. Oligotyping Analysis 

In total, 15 oligotypes were found for *Pyricularia* and 7 for *Bipolaris* that were identified by BLASTn as showed in the Supplementary Table S6.

The correlation between the number of significant oligotypes and their relative abundance for each day is represented in Supplementary Figure S6. Supplementary Figure S6A shows the number of oligotypes together with the relative abundance within the genus *Pyricularia*. In the case of *Pyricularia*, the number of oligotypes ranged from 6 at the beginning of the trial (second week) to 15 at the end of the trial. Whilst the genus *Pyricularia* showed a maximum abundance during the end of July and August, the number of oligotypes remained constant. The most abundant oligotypes were Pyr1, which was found in all the samples except for the second day of the trial, and Pyr2 and Pyr3, which were also present at high relative abundance in all the samples. The genus *Bipolaris* presented a more homogenous distribution of the seven oligotypes despite the abundance of the genus (2%) in all the samples. The least abundant oligotype was Bip7 (Supplementary Figure S6B).

According to the RDA analysis, the environmental variables and the phenological stages explained a 53.3% of the oligotyping relative abundance, however after adjusting this number to the number of variables, it was reduced to 41.3%. The triplot showed that the weather data affected each *Pyricularia* oligotype in a different way, distributing them as a function of temperature, wind speed, rainfall and RH (Supplementary Figure S7). The relative abundance of Pyr1 was highly influenced by high relative humidity, whilst Pyr2 and Pyr3 rose with high wind speed, and the relative abundance of Pyr7, Pyr8, and Pyr9 increased with higher rainfall.

The same rda analysis performed for the *Bipolaris* oligotypes, showed that the environmental parameters only explained 31.2% of the data (reduced to 13.7% after adjusting to the number of variables) (Supplementary Figure S8).

Regarding the correlation between *Pyricularia* oligotypes and temperature, two oligotypes (Pyr 4 and Pyr 9) varied in a significant way across the trial. Pyr 4 showed the highest abundance at temperature quartile T4 (2.5%) and the lowest one at T3 (1.1%). Pyr9 abundance decreased with increasing temperature, passing from 1.93% at T1 to 0.67% at T4. No significant influence (FDR > 0.05) of the temperature was shown in the *Bipolaris* oligotypes. 

However, the relative humidity influenced the relative abundance of Bip7 that changed from 3% to 0.58%, 0.46%, and 0.48% (FDR < 0.05). Relative humidity did not cause significant variations in the *Pyricularia* oligotypes if FDR was assessed, however the increase of relative humidity caused an increase in the relative abundance (*p*-values < 0.05) for Pyr1 (56.7%, 55.7%, 63.3%, 70.2%) and a reduction for Pyr2 (16.4%, 16.5%, 15.7%, 7.2%) Pyr7 (3.1%, 1.1%, 1.6%, 1.6%) and Pyr11 (0.5%, 0.65%, 0.5%, 0.5%). 

Pyr10 changed in a significant way with the wind speed, as the pairwise Wilcox test showed statistically significant differences between the third and fourth quartile (0.6% and 0.9% relative abundance, respectively). 

The results of the pairwise Wilcoxon tests also revealed that only some of the oligotypes analyzed varied in a significant way across the months: 10 oligotyping of *Pyricularia* (Pyr 2, 3, 4, 6, 9, 11, 12, 13, and 14) (Supplementary Figure S9) changed in a significant way, however, no effect was observed clustering the *Bipolaris* oligotypes as a function of months (FDR > 0.05). 

On the other hand, the same Pairwise Wilcox test revealed strong influence of the growth stages of rice in the oligotypes of *Pyricularia* (Supplementary Figure S10; FDR< 0.05) in Pyr1 (*P. grisea*, from 27.1% to 58.5%, 58.4%, 68.8%, 62.9%, 68%, 77%), Pyr2 (*P. oryzae*, from 32% to 15.5%, 8.9%, 12.4%, 16.2%, 10.2%, 3.4%), Pyr3 (*P. oryzae*, from 33.8% to 12.5%, 6.1%, 8%, 8.7%, 6.7%, 2.4%), Pyr6 (*P. grisea*, from 0.6% to 1.7%, 0.8%, 1.1%, 1.6%, 2.3%, 2.6%), and Pyr9 (*P. grisea*, from 0% to 0.6%, 0.4%, 0.6%, 1.1%, 2.9% and 4.9%). 

Similarly, for the oligotypes of *Bipolaris* there was a strong influence of the phenological phase of rice (Supplementary Figure S11): Bip1 (*Bipolaris* sp.) passed from 34.9% to 27.5%, 21.7%, 21.8%, 21.9%, 18.1%, and 21.9%; Bip2 (*Bipolaris* sp.) passed from 24.3% to 19.8%, 15.9%, 15.3%, 13.7%, 13.6%, and 18.2%, Bip3 (*Curvularia* sp.) passed from 11.8% to 15.7%, 18.8%, 19.8%, 20.5%, 20.5%, and 20.4%); Bip4 (*Curvularia* sp.) passed from 11% to 12.5%, 17.2%, 15.9%, 15.5%, 17.1%, and 17.4%; and Bip6 (*Curvularia* sp.) passed from 7.4% to 10.2%, 11.8%, 12.6%, 12.5%, 14.1%, and 9.7%. 

### 3.6. Correlation between Oligotypes and Number of Cells Calculated by qPCR

The oligotyping results reported the presence of at least two species of *Pyricularia* during the trial. Positive and negative correlation between the relative abundance calculated as explained above and the number of cells of *P. oryzae* were found. Supplementary Table S7 shows the results of Spearman’s rho value and the *p*-value of each oligotype. Strong positive relationships were found with the oligotypes Pyr1, Pyr4, Pyr5, Pyr6, Pyr7, Pyr8, Pyr9, Pyr10, Pyr11, Pyr12, Pyr13, and Pyr15 with rho >0.3%. The strongest positive relationship was found with Pyr9 identified as *P. grisea* which was strongly influenced by relative humidity and the plant growth stage. The oligotypes identified as *P. oryzae* (Pyr2 and Pyr3) and the oligotype identified as *P. grisea* (Pyr14) did not correlate in a significant way (*p*-value > 0.05) with the number of cells calculated with the TaqMan assay. No significant correlation (*p*-value > 0.05) was found between the oligotypes of *Bipolaris* and the number of cells calculated using the TaqMan assay. 

## 4. Discussion

A better understanding of the methods for recording the movements of fungal spores facilitates long-term monitoring, surveillance activities, and disease forecasting [54]. This study focused on the cropping season of rice, as a model, to evaluate the potential of high-throughput sequencing (HTS) technologies and oligotyping to study the airborne mycobiota. In particular, we focused on two important rice pathogens due to their widespread occurrence in all rice-growing countries [55]. The positive and negative correlations with other fungal species that may influence the dynamics of the habitat and may compete for space was included.

The Burkard spore trap was selected due to its highly efficient collection of spores in contrast with other devices [56,57]. The difficulty in the collection of the airborne particulate, the low amounts of organisms collected in the samples and the problematic DNA extraction are challenging steps for airborne-mycobiota studies [38,58,59], but they were successfully overcome in this study. 

In addition, daily sampling was adopted, to fulfil one of the requisites of [54], which is the need of continuous sampling to detect spores that may have travelled over long distances. Sampling could extend to all the year, but it is appropriate to reduce it to the key growing season to correlate the dynamics of inoculum with the disease assessment data and the biotic and abiotic stresses and to posteriorly perform modelling [54,60].

### 4.1. Rice Pathogen Monitoring

*P. oryzae* is an endemic pathogen in all rice-growing countries, which is favored by the climatic conditions of Mediterranean countries due to the alternation of wet and dry periods. The rice blast pathosystem has been divided into two subsystems, the leaf blast and the neck blast [61] treated separately, as their relationship is still unclear [31]. In addition, the pathogen is able to infect rice at any stage of the host life cycle. In this study, the first symptoms of leaf blast occurred in the field at BBCH 47 corresponding to the opening of flag leaf, while neck blast occurred later at BBCH 57 when over 70% of the panicles emerged. The presence of clear symptoms in this susceptible cultivar suggested an increase of the relative abundance of the air spores of *P. oryzae* causing secondary infections that spread the disease to other plants.

*Pyricularia* spp. was found throughout the rice cropping cycle with relative abundance ranging from 0.6% to 2%, though non-typical symptoms of rice blast were found in June. TaqMan qPCR demonstrated the presence of *P. oryzae* spores in the field and the maximum of 80,000 cells/day was reached in August. In Yeh and Bonman [62] and Castaño et al. [63], 80,000 cells were produced by the spikelet lesions, and 280,000 cells per neck node lesion were reported. However, they observed a differential amount of spores related to the age of the leaf reaching over 36,000 cells/day during the early dough with a maximum up to 92,000 cells/day, that may cause a higher number of leaf and neck lesions, which release spores disseminated over long distances. Initial symptoms of rice blast on leaves depends on the age of the plant, with leaf blast severity higher in young plants (3/4 leaf stage) than in older plants [64], and in our case the maximum was reached during the flag leaf opening stage (BBCH 47). Mature lesions can produce conidia when the relative humidity is higher than 89%, the temperature reaches 20 °C [31], and water droplets cause the spore movement from one plant to the other [65,66]. In this study, the disease index in the leaf and neck was correlated with temperature quartiles, and the symptoms were acute when the average daily temperature was lower than 24 °C. No significant correlation was found with the relative humidity using the disease index of neck and leaf blast, however the rda analysis suggested that influenced the relative abundance of this pathogen. 

Calvero et al. [67] and Suzuki [66] reported a negative correlation between leaf blast symptoms with wind speed ≥ 3.5 m/s. The average wind speed in our data was inferior to 2.9 m/s and the disease index of leaf and neck blast increased, especially, with wind ranging from 1.1 to 1.3 m/s. This range of wind speed could still be an advantage for increasing the movement of the air spores within the canopy, but it does not favour the movement over long distances.

Calvero et al. [67] and the modeling of Esmailpoor [68] and Mousanejad et al. [69] reported the presence of peaks of spores 3–5 days after rainfall and therefore bigger incidence of blast 7–10 days after optimal weather conditions. In the current study, the rda data suggested low rainfall events for increasing the relative abundance of *Pyricularia* and we observed the first increase in *P. oryzae* cells after three days of rainfall.

On the other hand, no brown spot symptoms were observed in the rice field. The optimal temperature for infection and lesion expansion corresponds with conidial germination (25–30 °C) and hyphal growth (27–30 °C) [70]. Despite the lack of correlation between temperature and brown spot development [71], an increase in the relative abundance of *Bipolaris* spp. was reported with higher temperatures. This information was obtained with the metadata but could not be confirmed using qPCR, due to the different taxonomical depth reached by the two techniques.

Drought could also positively affect the development of the brown spot symptoms [72]. However, in 2016, there were regular rainfall events during March in northern Italy (39 mm in contrast with the common average of 18 mm). From March to April, the rainfall events decreased, however a new increase during May to 50 mm of rainfall was registered. Taking into account that brown spot is usually correlated with limited rainfall, but high relative humidity [73], the climatic events of May could explain the absence of brown spot symptoms in the field and the absence of conidia detected by the TaqMan at the beginning of the trial during tillering, panicle initiation and boot stage. High humidity is the second factor favouring the development of brown spot, but relative humidity was quite low on average (73.6 ± 6.4%).

Padmanabhan and Ganguly [74] reported the importance of the age of the plant in the development of brown spot: younger plants show a lower infection rate, which tends to progressively increase with plant age. They also observed highest infection rates in plant inoculated in September, when the highest number of spores were captured and quantified using the qPCR assay. Usually 6 days-after infection is the highest sporulation peak of *B. oryzae* [75] and the time between latency and infection could range between 6 and 19 days [76]. 

In both cases, the qPCR was useful for quantifying the spore inoculum, although the repeatability was poor, probably due to the high abundance of other fungal genera in the samples and to the low amount of target pathogen. On the other hand, due to the artefacts caused by PCR bias, sequencing errors and artifacts or chimeras, the output of metabarcoding is not considered quantitative [77] and therefore the only data provided concern the relative abundance of the OTUs. As a consequence, if targeted and untargeted approaches are used to quantify microbial communities, they can be adopted to achieve different objectives.

### 4.2. Mycobiota Present in the Rice Paddy

Inflows and outflows of organisms to and from ecosystems, as well as their birth and death rates, regulate the dynamics of populations. One of the most important sources of spore inoculum, which is posteriorly transmitted by the air, is the phyllosphere [78]. The predominant fungal genera from the phyllosphere which are leaf age, season, variety, and environment dependent are *Alternaria*, *Aspergillus*, *Bipolaris*, *Chaetomium*, *Cladosporium*, *Curvularia*, *Fusarium*, *Memmoniela*, *Mycosphaerella*, *Setosphaeria*, *Stachybotrys*, *Aurebasidium*, *Phoma*, *Trichoderma*, and *Sporobolomyces* [79,80].

In this study, 64 OTUs were determined throughout the growing season. Previous studies about the biodiversity of fungal spores in paddy fields reported 40 fungal spore types [81], 42 fungal spores type [82], and 39 genera [83], including *Alternaria*, *Cercospora*, *Fusarium*, *Cladosporium*, and *Curvularia*. The Shannon index, as well as the overall number of OTUs, provide information about the reduction of biodiversity from June to September. This result may imply that some of the OTUs become predominant during the final stage of the crop production cycle when rice is ripening and therefore can be directly competing with the target pathogens. In particular, a significant correlation with the ripening stage was found for some saprophytic fungi, such as *Alternaria* or *Myrothecium*, and some pathogens, such as *Bipolaris*. 

By considering the most abundant OTUs, not all of them increased in their abundance during the trial, but they showed different behaviour. The relative abundance of some OTUs decreased during the trial, including *Epicoccum* and *Davidiella*, whilst other reached the maximum during July or August, probably as part of the colonization pattern of the fungi on the plants. The positive and negative correlations also gave an idea of the predominance of some fungi against others, showing competence between different genera for nutrients or space in the plant surface. The meteorological parameters did not affect all OTUs in the same way.

The results of our study indicate that the core mycobiota tend to dominate the air mycobiota and to exclude other taxa and this dynamic can affect the development of a disease by competence in the habitat. *Cladosporium* spp. was the most abundant genus throughout the cropping cycle. *Cladosporium* is an outdoor and indoor fungus [84], whose relative abundance increases in the summer with a reduction during the winter and a significant increase of spore release in August [85], which was confirmed by our results where the highest abundance was found during the warmest months, July and August. *Alternaria*, *Davidiella*, and *Epicoccum* were found during the trial, with a maximum at the beginning of the cropping cycle (June–July), confirming the result of Hollomon [79] who reported the genera as the most important in the phyllosphere in mid-July. 

*Myrothecium* reached up to 25% of relative abundance during July and August, when the daily temperature was higher (24.6 °C to 26.1 °C) and surprisingly correlated positively with the development of leaf and neck rice blast symptoms highlighting the importance of studying together the dynamics of both fungi. Most species of *Myrothecium* are saprophytes, though some are seedborne rice pathogens [86,87]. The positive correlation with rice blast symptoms showed here might be explained by similar ecological needs of both *P. oryzae* and *Myrothecium* spp. In addition, *Myrothecium* was demonstrated to be an invasive pathogen of leafy vegetables in Italy with an incidence growing year after year [88,89,90] and the potential to produce the mycotoxins verrucarin A and roridin E [91,92]. Only few species of *Myrothecium* have been until now characterized for their mycotoxicological profile, however, the correlation between climate conditions and macrocyclic trichothecene production was previously demonstrated especially at the high temperatures of 26–30 °C [92]. The higher relative abundance of *Myrothecium* could be a significant source of mycotoxins, which should be considered by the food industry. 

### 4.3. Oligotyping

Although ITS is the described as the universal barcode for fungi, issues can arrive due to the presence of intraspecific variation in some nucleotide-positions caused by the high number of ITS copies in a single eukariotic genome (between 30 and 30,000) [93]. The theory of the concerted evolution describes the homogenization of multicopy gene sequences over time, resulting in low variation within species or individuals [94,95]. However, some gene families, such as 5S RNA, present an evolutionary process called birth and death evolution, where homogenization does not occur [96]. Simon and Weiss [97], in a comprehensive examination of the polymorphisms of three nuclear ribosomal loci, i.e., SSU, LSU, and ITS, reported a high level of unexpected single nucleotide polymorphisms in important plant pathogenic ascomycetes *Mycosphaerella punctiformis*, *Davidiella tassiana*, *Teratosphaeria microspora*, and *Phoma exigua* var. *exigua* contrasting with the idea of concerted evolution. The robustness of the ITS region is helpful to determine the phylogenetic relationship of not closely related species which is useful to determine the mycobiota present at genus level. However, at a lower level, the implications of the intragenomic variation on the ITS sequence may cause conflicts on the phylogenetics, ecology, and diagnostics of diseases. 

Oligotyping analysis has been used to reach a sub-OTU level in order to explore a possible new pipeline useful for analysis of air spores. Based on our knowledge, this type of analysis has not been used for fungal studies until now. Oligotyping is able to detect a higher number of sub-OTU populations than the OTU clustering approach. Each OTU can be split into several distinct subpopulations that differ for few nucleotides in the sequence [53].

In the case of *Pyricularia* spp. and *Bipolaris* spp., other barcodes could be more appropriate to reach a species level identification. However, the strong correlation between the output of the qPCR and the different *Pyricularia* oligotypes, which were identified as *P. oryzae* and *P. grisea*, demonstrated the high reliability of this analysis, even with fungi. 

However, the number of cells of *B. oryzae* was not correlated with any of the oligotypes of *Bipolaris* identified as different taxonomical groups. No oligotype identified as *Bipolaris* was obtained due to the filtering of the pipeline, showing a limit of detection for this approach. However, the combination with the TaqMan assay could solve this problem. 

## 5. Conclusions

Temperature, relative humidity, wind speed, and growth stage of the plant affect the composition of the dominant mycobiota at genus level but also have a strong effect at the sub-genus level. This confirmed the effects in the epidemiology of both pathogens. 

In this study, we described the use of two molecular techniques that provide information relevant to biotic stress and can be perfectly combined with environmental observation and weather forecasting to develop new disease models and therefore a disease support system (DSS). The components of a DSS should be integrated to generate the most appropriate model, including observation of symptoms and crop growth-stage, biotic stress that can be caused by spore pressure and dynamics in the air, abiotic stresses (weather forecasting), and the microclimate of the region or area. Pesticide treatment schemes rely on forecasting models based on meteorological conditions favouring disease development. The data presented here include a correlation with the meteorological conditions during the growing season, but also help to understand the fungal genera correlation and exclusion pattern and the dynamics within the air-paddy field system over time. Taking into account that the occurrence is positively correlated with the inoculum amount, and that we demonstrated the successful use of metabarcoding, oligotyping, and qPCR approaches. These techniques may be used for evaluating the progression of aerial spore occurrence during the season. The use of more than one spore-trap device in vast areas might be necessary to understand the movement of spores over long distances. Aerial mycobiota is a factor which should be included in disease forecasting modelling, to help growers in the decision making process about crop protection strategies and timely application of fungicides. The sucessful identification of both rice blast and brown spot agents using HTS might open the door to future applications to monitor pathogens and reduce the risk of disease outbreaks. The current bottlenecks in using HTS in the field for pathogens remain the cost of the analysis and the need for expertise in data interpretation.

## Supplementary Materials

The supplementary are available online at https://www.mdpi.com/2309-608X/6/4/372/s1.
