# Supplementary material for: Monitoring and Surveillance of Aerial Mycobiota of Rice Paddy through DNA Metabarcoding and qPCR
Source: J Fungi (Basel). 2020 Dec 17;6(4):372. doi: 10.3390/jof6040372 (PMC7766667; doi:10.3390/jof6040372)
Supplement: Supplementary file 1 [file jof-06-00372-s001.pdf]

Journal of *Fungi*

MDPI

Article

Monitoring and surveillance of aerial mycobiota of rice paddy through DNA metabarcoding and qPCR

Supplementary Materials:

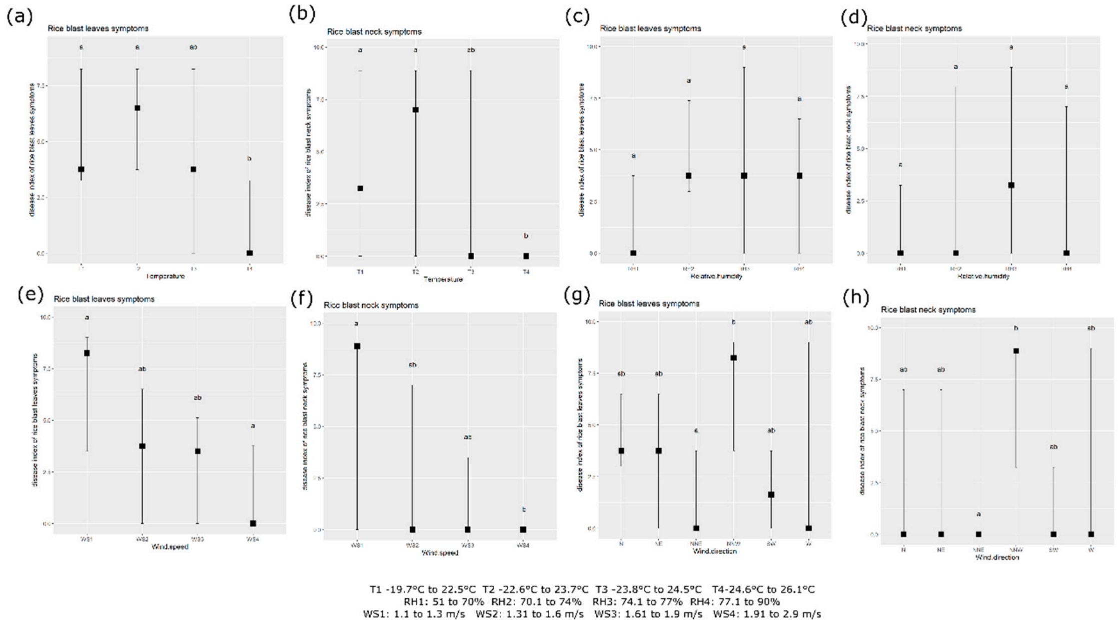

Figure S1. Representation of the leaves (a, c, e, g) and neck (b, d, f, h) blast symptoms index and the weather data. A and B. Kruskal-Wallis rank sum with Dunn test of the groups determined by the temperature quartiles. C and D: Kruskal-Wallis rank sum with Dunn of the groups determined by the relative abundance quartiles. E and F: Kruskal-Wallis rank sum with Dunn of the groups determined by the wind speed quartiles. G and H Kruskal-Wallis rank sum with Dunn of the groups determined by the wind direction.

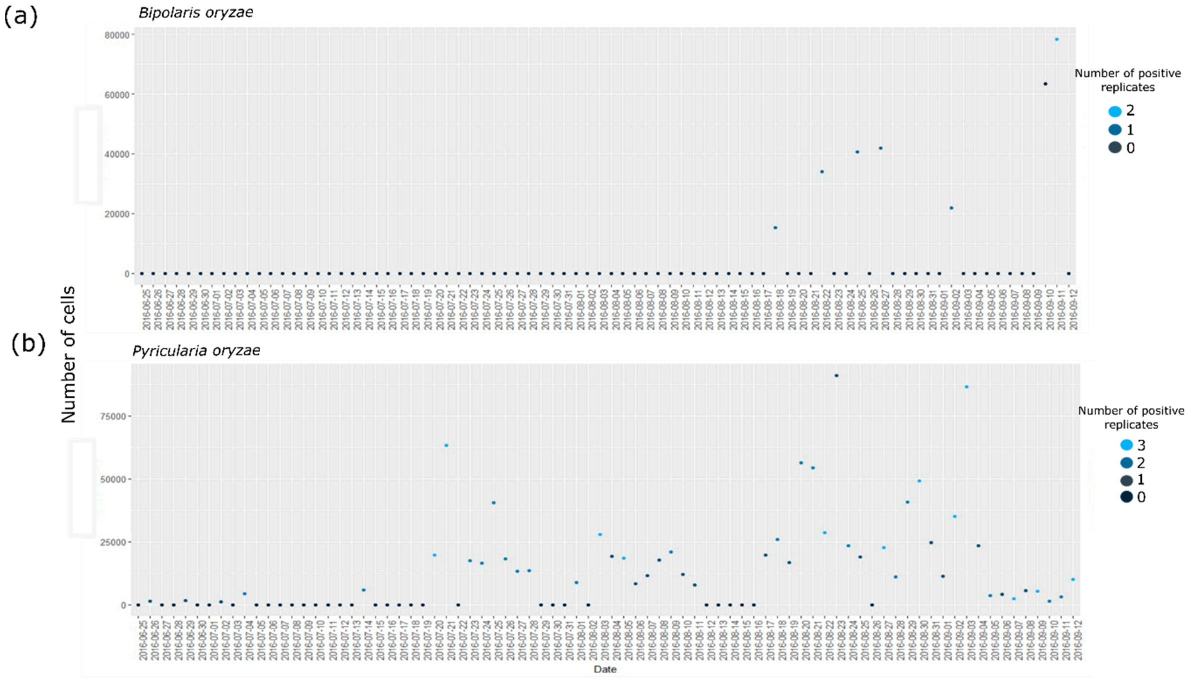

Figure S2. Number of cells of *Bipolaris oryzae* (a) and *Pyricularia oryzae* (b) present among the trial calculated according the Ct of the qPCR assays.

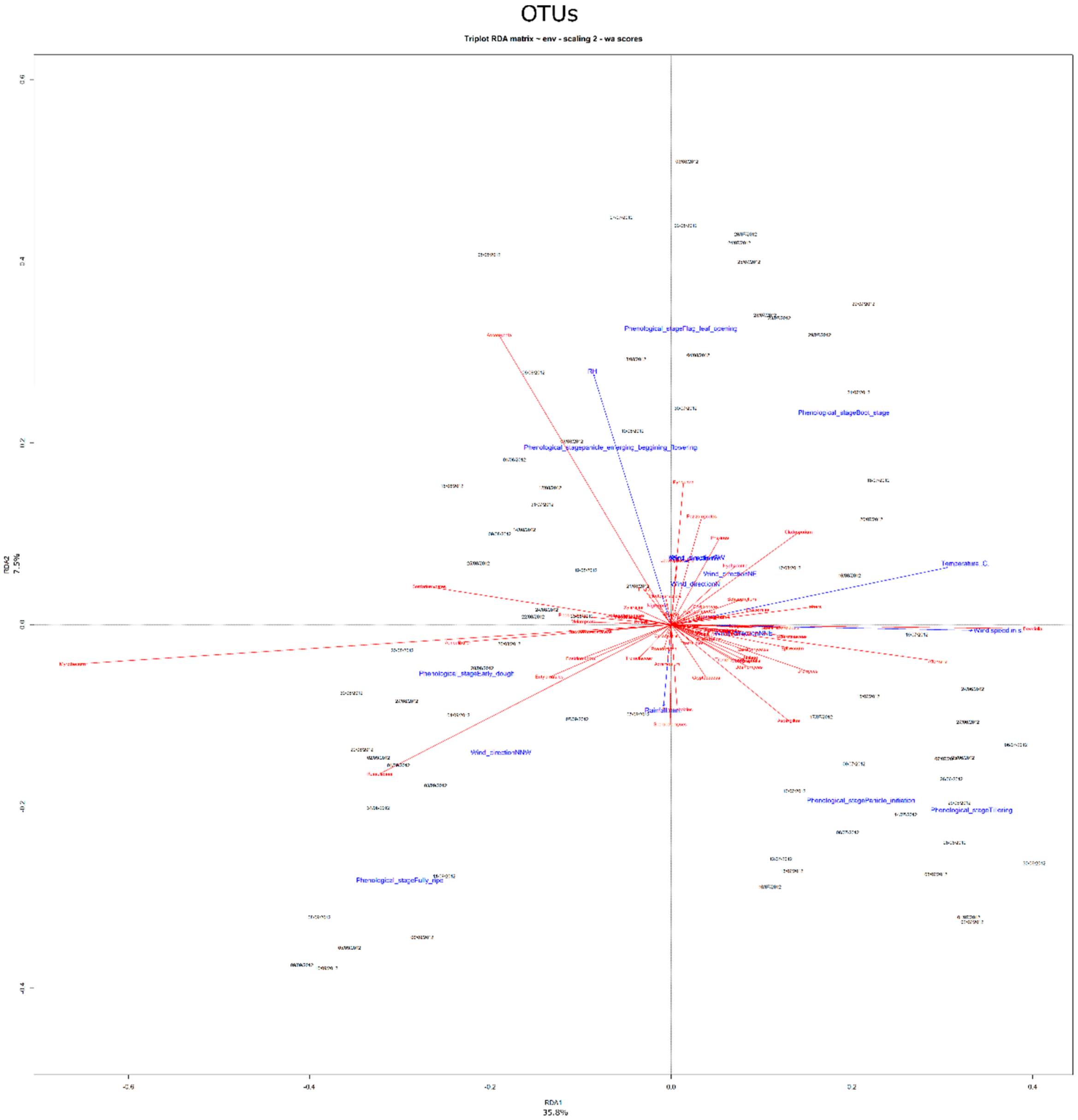

Figure S3. Redundancy Analysis (RDA) representing the Axis 1 and 2 among the OTUs and the weather data and phenological stages. Proportion explained by two first constrained ordinations are reported as percentages in the axes.

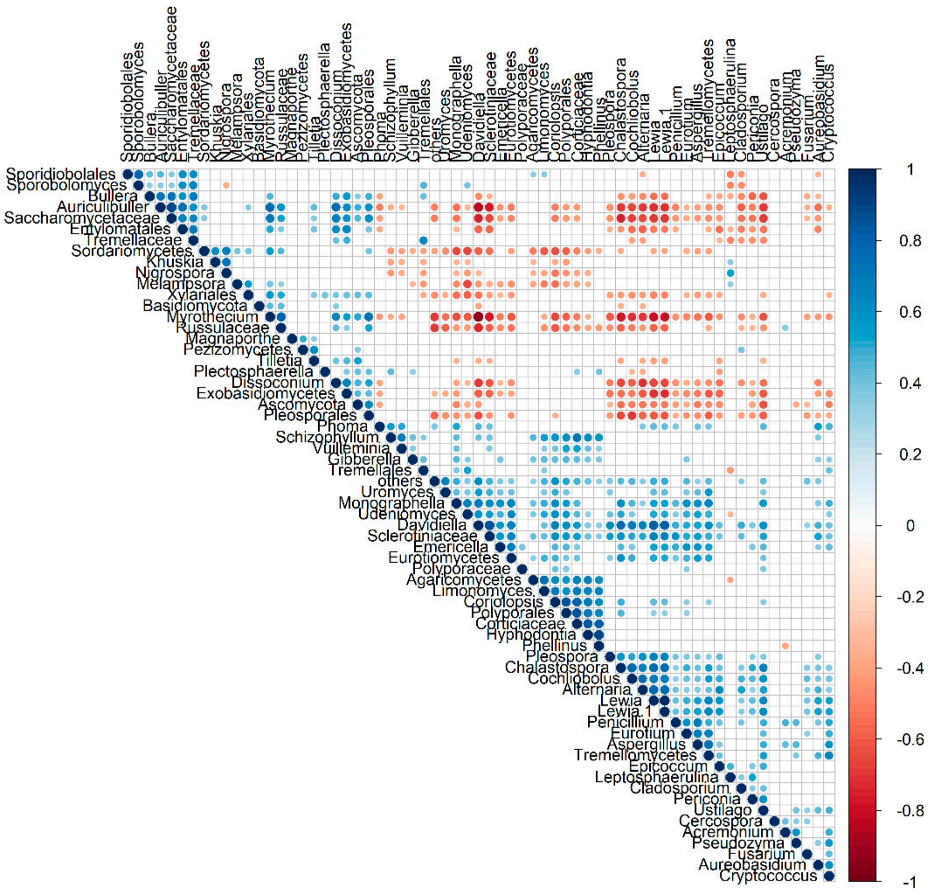

Figure S4. Significant co-occurrence and co-exclusion relationships between species. Spearman's rank correlation matrix of the genera identified with > 0.2% abundance in at least 20 samples. The plots only represent significant differences FDR < 0.01. Strong correlations are indicated by large circles, whereas weak correlations are indicated by small squares. The colour of the scale bar denotes the nature of the correlation, with 1 indicating perfectly positive correlation (dark blue) and -1 indicating a perfectly negative correlation (dark red).

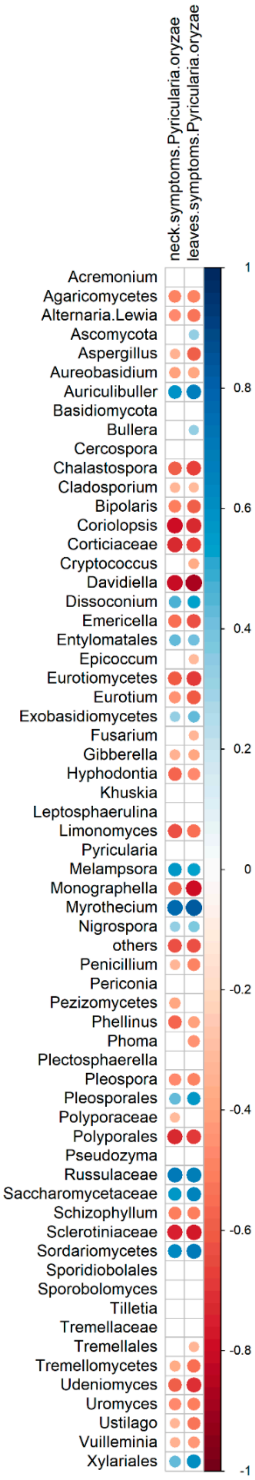

Figure S5. Significant correlation between OTUs and the *Pyricularia* leaves and neck symptoms using Spearman's rank correlation matrix of the genera identified with > 0.2% abundance in at least 20 samples. The plots only represent significant differences FDR < 0.01. Strong correlations are indicated by large circles, whereas weak correlations are indicated by small circles. The colour of the scale bar denotes the nature of the correlation, with 1 indicating perfectly positive correlation (dark blue) and -1 indicating a perfectly negative correlation (dark red).

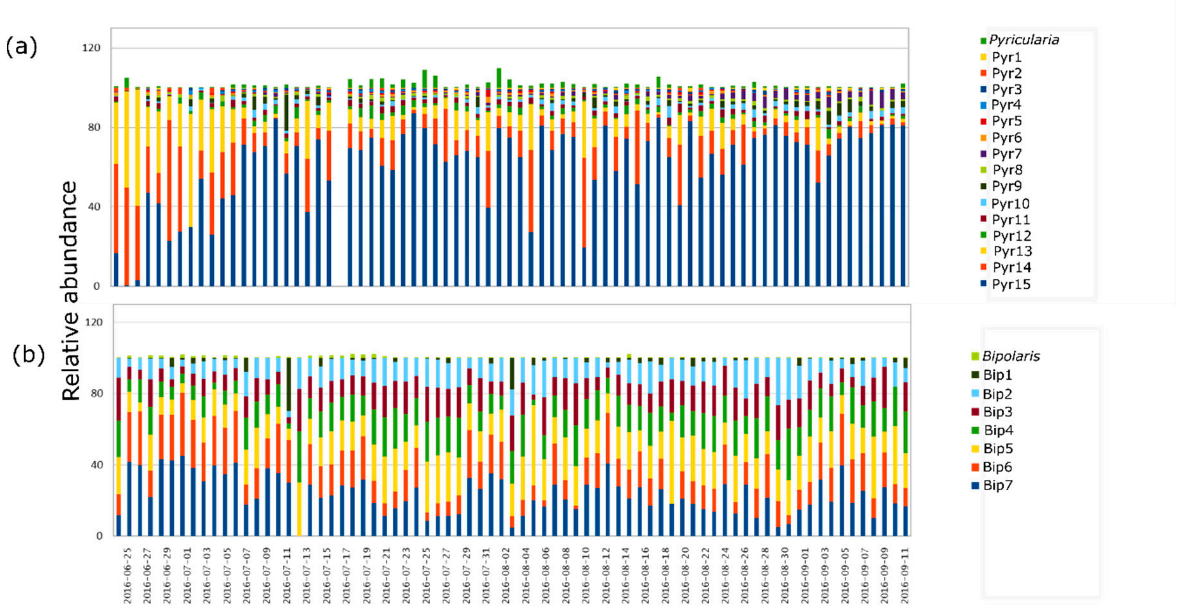

Figure S6. Distribution of the oligotypes of *Pyricularia* (a) and *Bipolaris* (b) over time.

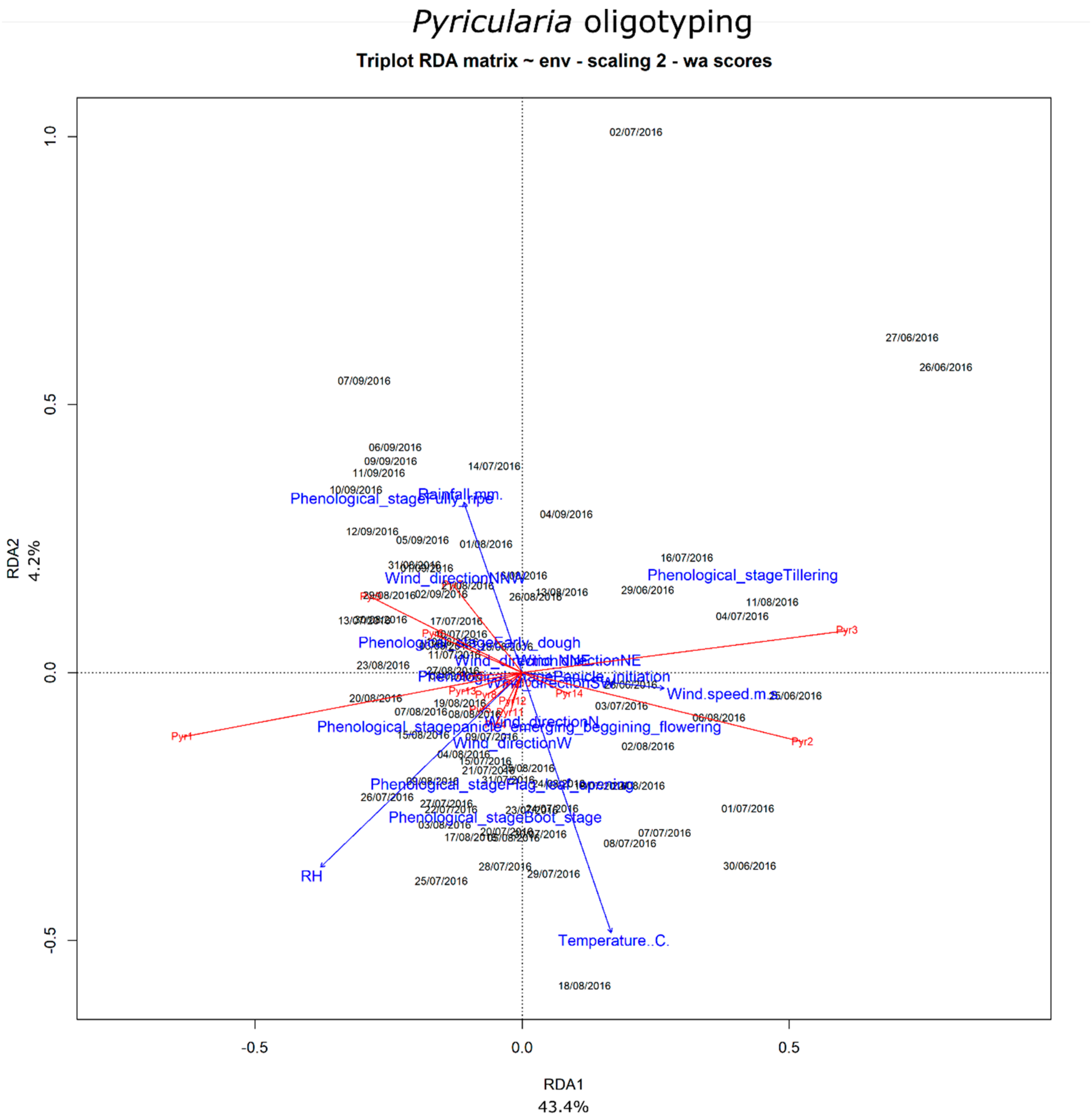

Figure S7. Redundancy Analysis (RDA) representing the Axis 1 and 2 among the *Pyricularia*

oligotypes and the weather data and phenological stages. Proportion explained by two first constrained ordinations are reported as percentages in the axes.

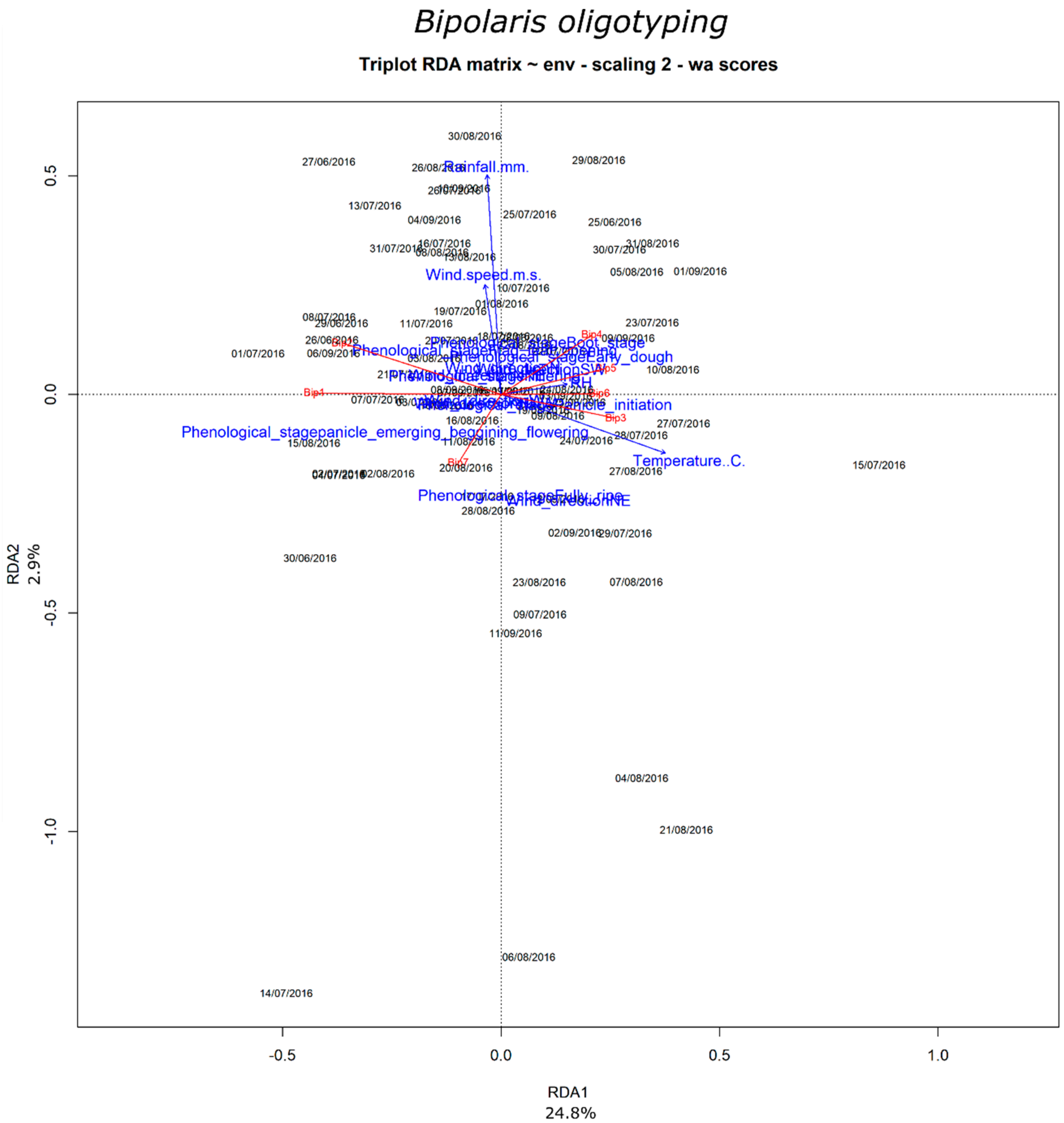

Figure S8. Redundancy Analysis (RDA) representing the Axis 1 and 2 among the *Bipolaris* oligotypes and the weather data and phenological stages. Proportion explained by two first constrained ordinations are reported as percentages in the axes.

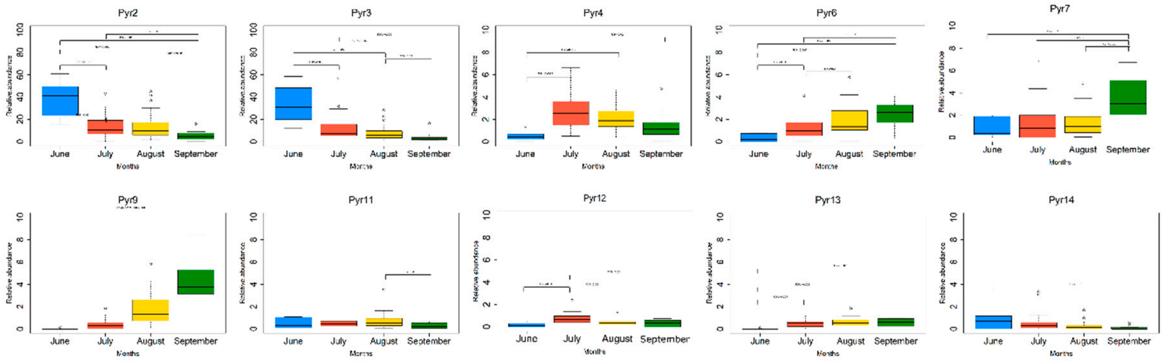

Figure S9. Abundance (%) of *Pyricularia* oligotypes significantly different (FDR < 0.05) among the sampling month (June, July, August and September).

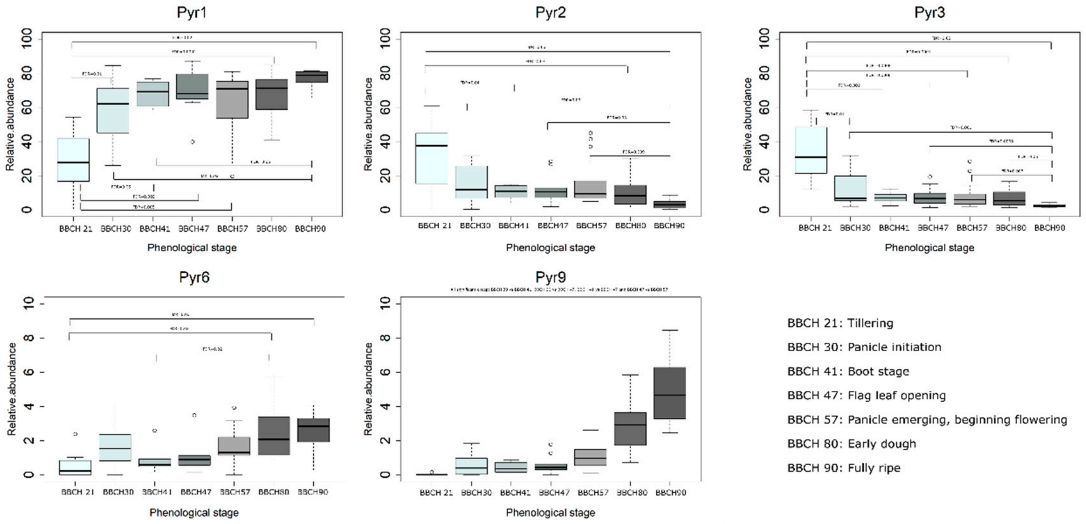

Figure S10. Abundance (%) of *Pyricularia* oligotypes significantly different (FDR < 0.05) among the growth stages (BBCH 21: tillering; BBCH 30: panicle initiation; BBCH 41: boot stage; BBCH 47: leaf opening; BBCH 57: panicle emerging; BBCH 80: early dough; and BBCH 90: fully ripening).

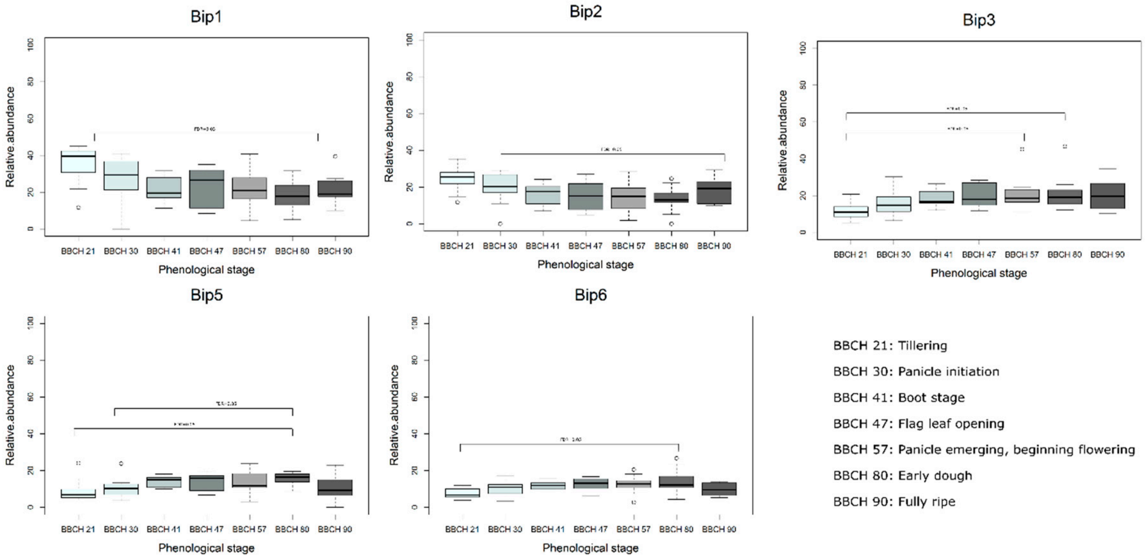

Figure S11. Abundance (%) of *Bipolaris* oligotypes significantly different (FDR < 0.05) among the growth stages (BBCH 21: tillering; BBCH 30: panicle initiation; BBCH 41: boot stage; BBCH 47: leaf opening; BBCH 57: panicle emerging; BBCH 80: early dough; and BBCH 90: fully ripening).

Table S1. Growth stages of the rice paddy during the trial.

DATA	PHENOLOGICAL STAGE	IDENTIFICATION KEYS OF RICE
**07/05/2016**	Sowing in water	
**14/05/2016**	BBCH:10-11	Imperfect leaf unrolled, tip of first true leaf visible- First leaf unfolded
**30/05/2016**	BBCH:13-14	3 leaves unfolded
**14/06/2016**	BBCH:21	Beginning of tillering: first tiller detectable
**21/06/2016**	BBCH:22-23	2-3 tillers detectable
**05/07/2016/**	BBCH:30-34	Panicle initiation or green ring stage: chlorophyll accumulates in the stem tissue, forming a green ring-Internode elongation or jointing stage: internodes begin to elongate, panicle more than 2 mm long (variety-dependent)
**19/07/2016**	BBCH:41	Early boot stage: upper part of stem slightly thickened, sheath of flag leaf about 5 cm out of penultimate leaf sheath
**26/07/2016**	BBCH:47-49	Flag leaf sheath opening-open
**04/08/2016**	BBCH:57-61	70% of panicle emerged- Beginning of flowering: anthers visible at top of panicle
**20/08/2016**	BBCH:80	Early dough
**05/09/2016**	BBCH:90	Fully ripe: grain hard, difficult to divide with thumbnail

Table S2. Meteorological conditions during the trial in the rice paddy.

Date	**Rainfall (mm)**	*Temperature (°C)*	*Relative Humidity (%)*	*Wind Speed (m/s)*	*Wind Direction*
25/06/2016	0	24.3	73	2.8	NNE
26/06/2016	0	24.4	71	1.9	NE
27/06/2016	0	24	66	1.6	N
28/06/2016	0	23.6	69	2.0	NNE
29/06/2016	0	24.8	67	2.0	NNE
30/06/2016	0.2	24.5	76	1.6	W
01/07/2016	0.4	25	71	1.3	N
02/07/2016	0	23.8	81	1.8	NNE
03/07/2016	11.4	25.1	75	1.2	N
04/07/2016	0	23.3	74	2.2	NE
07/07/2016	0	25.1	77	1.7	N
08/07/2016	0	26.1	74	2.1	NE
09/07/2016	0	27.7	69	1.2	SW
10/07/2016	0	27.5	72	1.7	NNE
11/07/2016	0	26.1	78	2.2	NNE
13/07/2016	13.6	21.8	78	2.4	N
14/07/2016	15.2	19.7	63	2.5	NNW
15/07/2016	0.4	20.3	51	2.9	NNE
16/07/2016	0	21.9	59	1.6	SW
17/07/2016	0	22.8	68	1.8	NNE
18/07/2016	0	24.6	68	1.5	NNW
19/07/2016	0	25.0	72	1.2	N
20/07/2016	0	26.7	70	1.6	N
21/07/2016	0	26.0	69	1.6	NNE
22/07/2016	0.6	22.5	88	2.0	N
23/07/2016	2.2	21.5	88	1.9	W
24/07/2016	0.2	24.1	76	1.2	W

25/07/2016	0	25.1	78	1.2	W
26/07/2016	0	26.4	72	2.1	NNE
27/07/2016	0	24.1	79	2.2	N
28/07/2016	0.6	24.5	74	1.3	SW
29/07/2016	0	25.1	76	1.7	N
30/07/2016	0	26.3	74	1.4	N
31/07/2016	0	23.0	87	2.1	NNE
01/08/2016	19.6	24.0	78	1.7	NE
01/08/2016	19.6	24.0	78	1.7	NE
02/08/2016	0	23.8	74	1.8	NNE
03/08/2016	0	24.4	81	1.4	N
04/08/2016	0	25.1	77	2.0	NE
05/08/2016	2	21.1	81	2.2	NNE
06/08/2016	1	22.3	68	1.5	N
07/08/2016	0	22.1	71	1.5	N
08/08/2016	0	22.9	71	1.3	NNW
09/08/2016	0	23.0	77	1.8	NNE
09/08/2016	0	23.0	77	1.8	NNE
10/08/2016	3.8	22.2	65	1.8	NNE
11/08/2016	4.4	19.9	66	1.8	N
13/08/2016	0	21.2	75	1.3	NNW
15/08/2016	0	24.5	72	1.4	N
16/08/2016	0	25.0	69	1.6	SW
17/08/2016	0	23.7	74	1.3	N
18/08/2016	0	23.6	76	1.6	N
19/08/2016	0	23.3	79	1.7	NE
20/08/2016	0	22.4	91	1.8	NE
21/08/2016	11	23.4	76	1.6	NNE
22/08/2016	0	21.1	66	1.9	NNW

23/08/2016	0	21.3	73	1.3	NNW
24/08/2016	0	22.2	73	1.3	N
25/08/2016	0	23.7	72	1.4	NE
26/08/2016	0	23.6	74	1.3	NNW
27/08/2016	0	23.6	74	1.3	NNW
27/08/2016	0	23.4	73	1.5	NNW
28/08/2016	0	24.5	72	1.4	N
29/08/2016	0	24.6	75	1.4	NNW
30/08/2016	3.4	21.3	87	2.2	N
31/08/2016	2	23.1	78	1.3	NNW
01/09/2016	0	23.9	75	1.1	W
02/09/2016	0	23.8	77	1.3	N
03/09/2016	0	23.9	78	1.2	N
04/09/2016	0	23.8	79	1.2	N
05/09/2016	0	23.4	70	1.6	NNE
06/09/2016	0	22.5	62	2.2	NNW
07/09/2016	0	21.2	68	1.8	NNW
08/09/2016	0	22.3	70	1.3	N
09/09/2016	0	22.5	74	1.2	NNW
10/09/2016	0	23.0	70	1.3	NNW
11/09/2016	0	22.7	75	1.1	NNW
12/09/2016	0	22.8	75	1.1	W

Table S3. Rice blast severity, indicated as disease index from 0 to 9, measured in the field during the cropping season (2016).

	19th July	*26th July*	*2nd August*	*10th August*	*17th August*	*23rd August*	*30th August*
NECK	0.0	0.0	0.0	3.2	7.0	8.9	9.0
LEAVES	3.0	3.7	4.0	3.2	6.5	8.2	9.0

Table S4. Alpha diversity values over time.

DATE	MONTH	SHANNON	CHAO1	OBSERVED OTUS	GOODS COVERAGE	ESC %
**25/06/2016**	June	5.728	1458.127	894.000	0.974	97.423
**26/06/2016**	June	5.657	1140.546	688.000	0.981	98.082
**27/06/2016**	June	6.256	1372.420	867.333	0.977	97.724
**28/06/2016**	June	5.390	1362.449	779.667	0.977	97.691
**29/06/2016**	June	5.211	1228.353	704.000	0.979	97.916
**30/06/2016**	June	5.455	1011.506	648.667	0.983	98.255
**01/07/2016**	July	4.765	1149.141	641.333	0.980	98.020
**02/07/2016**	July	5.362	1417.719	777.000	0.976	97.564
**03/07/2016**	July	5.014	1016.376	614.333	0.983	98.300
**04/07/2016**	July	4.900	1353.234	721.333	0.976	97.624
**07/07/2016**	July	4.494	1032.670	603.667	0.982	98.234
**08/07/2016**	July	5.091	1287.180	753.000	0.977	97.737
**09/07/2016**	July	5.631	949.915	560.333	0.986	98.588
**10/07/2016**	July	4.989	966.985	586.667	0.984	98.411
**11/07/2016**	July	5.132	1338.811	708.667	0.977	97.714
**13/07/2016**	July	5.402	1074.169	647.000	0.982	98.199
**14/07/2016**	July	5.255	1140.763	659.667	0.981	98.084
**15/07/2016**	July	4.734	861.833	544.000	0.986	98.648
**16/07/2016**	July	5.493	1156.065	725.333	0.982	98.178
**17/07/2016**	July	5.556	963.686	597.000	0.986	98.563
**18/07/2016**	July	5.304	1140.787	651.667	0.980	97.970
**19/07/2016**	July	4.390	1003.993	568.667	0.983	98.290
**20/07/2016**	July	4.871	889.239	533.500	0.986	98.571
**21/07/2016**	July	4.445	939.747	524.333	0.984	98.429
**22/07/2016**	July	4.458	918.721	547.333	0.985	98.500
**23/07/2016**	July	4.894	907.367	545.333	0.985	98.500
**24/07/2016**	July	5.704	1515.070	861.667	0.974	97.364
**25/07/2016**	July	5.078	765.592	441.667	0.990	98.956
**26/07/2016**	July	4.558	967.513	510.333	0.983	98.348
**27/07/2016**	July	5.140	731.388	431.667	0.989	98.875
**28/07/2016**	July	5.063	698.921	389.333	0.990	99.008
**29/07/2016**	July	5.061	1393.957	708.000	0.977	97.707
**30/07/2016**	July	4.595	1070.864	586.667	0.981	98.130
**31/07/2016**	July	5.540	873.635	534.000	0.987	98.727
**08/08/2016**	August	4.853	1148.918	537.333	0.981	98.126
**09/08/2016**	August	5.107	1032.232	566.000	0.983	98.259
**10/08/2016**	August	5.047	837.209	524.333	0.987	98.665
**11/08/2016**	August	5.544	1667.186	918.667	0.970	97.027
**13/08/2016**	August	5.173	968.854	540.667	0.984	98.377

**15/08/2016**	August	5.029	828.607	484.000	0.987	98.729
**16/08/2016**	August	5.127	1272.575	698.667	0.978	97.797
**17/08/2016**	August	4.534	827.134	454.667	0.986	98.627
**18/08/2016**	August	4.719	1231.400	614.500	0.979	97.906
**19/08/2016**	August	4.604	1197.655	549.000	0.980	98.038
**20/08/2016**	August	4.776	769.431	444.000	0.988	98.764
**21/08/2016**	August	4.597	1220.272	543.333	0.980	97.991
**22/08/2016**	August	4.619	1586.372	729.667	0.974	97.393
**23/08/2016**	August	4.602	1099.142	532.000	0.981	98.126
**24/08/2016**	August	5.028	1372.590	696.667	0.976	97.620
**25/08/2016**	August	4.582	1319.198	565.000	0.979	97.916
**26/08/2016**	August	4.401	1009.029	496.000	0.983	98.298
**27/08/2016**	August	4.715	1169.571	570.833	0.980	98.009
**28/08/2016**	August	4.592	1069.230	536.500	0.981	98.125
**29/08/2016**	August	4.651	1158.798	508.500	0.982	98.218
**30/08/2016**	August	4.183	1034.337	453.667	0.984	98.365
**31/08/2016**	August	4.917	1185.976	559.000	0.981	98.072
**01/08/2016**	August	5.211	1330.252	700.667	0.978	97.766
**02/08/2016**	August	4.899	1110.422	583.333	0.981	98.090
**03/08/2016**	August	5.016	831.902	412.667	0.989	98.870
**04/08/2016**	August	4.718	551.565	336.000	0.992	99.193
**05/08/2016**	August	5.079	1675.116	836.667	0.971	97.098
**06/08/2016**	August	5.584	1700.250	907.333	0.970	97.009
**07/08/2016**	August	5.394	1098.419	624.667	0.982	98.186
**01/09/2016**	September	4.516	1081.610	498.667	0.982	98.246
**02/09/2016**	September	4.582	1351.799	571.667	0.978	97.837
**03/09/2016**	September	4.175	1142.402	488.000	0.982	98.167
**04/09/2016**	September	4.955	1566.092	741.667	0.973	97.337
**05/09/2016**	September	4.422	1400.603	599.000	0.978	97.751
**06/09/2016**	September	4.554	1239.060	587.667	0.979	97.905
**07/09/2016**	September	5.148	1460.947	679.000	0.975	97.514
**08/09/2016**	September	4.908	1372.419	658.667	0.977	97.683
**09/09/2016**	September	4.336	1215.225	503.333	0.981	98.076
**10/09/2016**	September	4.539	1095.600	504.000	0.982	98.188
**11/09/2016**	September	3.857	985.095	437.667	0.984	98.388
**12/09/2016**	September	4.731	1587.578	657.333	0.975	97.525

Table S5. Average relative abundance (%) of the OTUs throughout the season.

OTU	Relative abundance (%)	OTU	Relative abundance (%)
Cladosporium	20.177	*Gibberella*	0.334
Alternaria	9.88	*Ustilago*	0.269

Myrothecium	7.312	*Pleospora*	0.231
Ascomycota	7.026	*Acremonium*	0.225
Epicoccum	5.787	Saccharomycetaceae	0.223
Basidiomycota	5.292	*Aureobasidium*	0.204
Sporidiobolales	5.154	*Tilletia*	0.195
Sordariomycetes	4.448	Exobasidiomycetes	0.174
Davidiella	4.299	Agaricomycetes	0.164
Russulaceae	2.727	*Dissoconium*	0.15
Leptosphaerulina	2.333	*Emericella*	0.147
Entylomatales	1.852	*Periconia*	0.144
Pyricularia	1.574	*Eurotium*	0.142
Auriculibuller	1.516	*Vuilleminia*	0.134
Pleosporales	1.357	Polyporaceae	0.134
others	1.332	*Pseudozyma*	0.13
Sporobolomyces	0.975	*Plectosphaerella*	0.13
Tremellaceae	0.88	*Penicillium*	0.13
Lewia	0.673	Tremellales	0.124
Bipolaris	0.568	*Limonomyces*	0.12
Uromyces	0.544	Eurotiomycetes	0.118
Hyphodontia	0.527	Tremellomycetes	0.115
Fusarium	0.516	Xylariales	0.115
Polyporales	0.502	*Chalastospora*	0.115
Schizophyllum	0.497	*Udeniomyces*	0.11
Coriolopsis	0.488	*Bullera*	0.109
Aspergillus	0.46	*Nigrospora*	0.101
Sclerotiniaceae	0.451	*Monographella*	0.096
Pezizomycetes	0.412	*Cercospora*	0.094
Melampsora	0.388	*Phoma*	0.094
Phellinus	0.346	*Khuskia*	0.086
Cryptococcus	0.339	Corticiaceae	0.078

Table S6. Identification of the oligotypes of *Pyricularia* and *Bipolaris* showing the BLASTn results the percentage of similarity and the GenBank accession numbers of the most similar sequences.

OLIGOTYPING	*BLAST RESULTS*	*SIMILARITY*	*GENBANK ACCESSION NUMBER*
**Bip1**	*Bipolaris austrostipae/Bipolaris cynodontis/Bipolaris coffeana*	99%	NR_147491/ HF934930/ HE792944
**BIP2**	*B. austrostipae/B. cynodontis/B. coffeana*	100%	NR_147491/ HF934930/ HE792944
**BIP3**	*Curvularia buchloes/Curvularia hawaiiensis/Curvularia spicifera/Bipolaris buchloes/Bipolaris maydis/Curvularia australiensis/Cochliobolus* sp./*Curvularia* sp.	99%	NR_147487/ HG778990/ HG778989/ HG778989/ JN207242/ HF934914/ KJ909765/ KJ913699/ KC986957/ JN207283/ JN207258/ FJ235087/ JN207242
**BIP4**	*C. buchloes/C. hawaiiensis/C. spicifera/B. buchloes/B. maydis/C. australiensis/Cochliobolus* sp./*Cochliobolus* sp.	100%	NR_147487/ HG778990/ HG778989/ HG778989/ JN207242/ HF934914/ KJ909765/ KJ913699/ KC986957/ JN207283/ JN207258/ FJ235087/ JN207242
**BIP5**	*C. buchloes/C. hawaiiensis/C. spicifera/B. buchloes/B. maydis/C. australiensis/Cochliobolus* sp./*Cochliobolus* sp.	100%	NR_147487/ HG778990/ HG778989/ HG778989/ JN207242/ HF934914/ KJ909765/ KJ913699/ KC986957/ JN207283/ JN207258/ FJ235087/ JN207242
**BIP6**	*C. buchloes/C. hawaiiensis/C. spicifera/B. buchloes/B. maydis/C. australiensis/Cochliobolus* sp./*Cochlibolus* sp.	100%	NR_147487/ HG778990/ HG778989/ HG778989/ JN207242/ HF934914/ KJ909765/ KJ913699/ KC986957/ JN207283/ JN207258/ FJ235087/ JN207242
**BIP7**	*Curvularia inaequalis*	99%	KX674660
**PYR1**	*Magnaporthe grisea*	99%	DQ493955
**PYR2**	*Pyricularia oryzae*	99%	KM816794
**PYR3**	*P .oryzae*	99%	KM816794
**PYR4**	*M. grisea*	99%	DQ493955
**PYR5**	*M. grisea*	99%	DQ493955
**PYR6**	*M. grisea*	99%	DQ493955
**PYR7**	*P. oryzae/P. grisea*	99%	JQ747492/ JF414841
**PYR8**	*M. grisea*	99%	DQ493955

**PYR9**	*M. grisea*	99%	DQ493955
**PYR10**	*P. oryzae*	99%	KM816794
**PYR11**	*P. oryzae*	99%	KM816794
**PYR12**	*M .grisea*	99%	DQ493955
**PYR13**	*M. grisea*	99%	DQ493955
**PYR14**	*M. grisea*	99%	FN555109
**PYR15**	*M. grisea*	99%	DQ493955

Table S7. Correlation of Spearman between the *Pyricularia* oligotypes and the number of cells calculated with a qPCR with TaqMan probe designed by Su'udi et al. 2013. The table reports the rho value and the p-value of each correspondence.

OLIGOTYPE	IDENTIFICATION WITH BLASTN	RHO	P-VALUE
**Pyr1**	*Magnaporthe grisea*	**0.47**	1.76e-05
**Pyr2**	*Pyricularia oryzae*	0.16	0.18
**Pyr3**	*P. oryzae*	0.08	0.49
**PYR4**	*M. grisea*	0.42	0.0002
**PYR5**	*M. grisea*	0.46	3.13e-05
**PYR6**	*M. grisea*	0.39	0.0006
**PYR7**	*P. oryzae/M. grisea*	0.30	0.009
**PYR8**	*M. grisea*	0.49	7.592e-06
**PYR9**	*M. grisea*	0.56	1.342e-07
**PYR10**	*P. oryzae*	0.33	0.003
**PYR11**	*P. oryzae*	0.38	0.0007
**PYR12**	*M. grisea*	0.36	0.001
**PYR13**	*M. grisea*	0.47	2.117e-05
**PYR14**	*M. grisea*	0.17	0.15
**PYR15**	*M. grisea*	0.48	1.174e-05

Publisher's Note: MDPI stays neutral with regard to jurisdictional claims in published maps and institutional affiliations.

© 2020 by the authors. Submitted for possible open access publication under the terms and conditions of the Creative Commons Attribution (CC BY) license (http://creativecommons.org/licenses/by/4.0/).
